# HCV Envelope protein 2 sequence comparison of Pakistani isolate and *In-silico* prediction of conserved epitopes for vaccine development

**DOI:** 10.1186/1479-5876-11-105

**Published:** 2013-04-30

**Authors:** Sobia Idrees, Usman A Ashfaq, Saba Khaliq

**Affiliations:** 1 Human Molecular Biology Group, Department of Bioinformatics and Biotechnology, Government College University (GCU), Faisalabad, Pakistan; 2 Department of Immunology, University of Health Sciences, Lahore, Pakistan

**Keywords:** HCV, E2 protein, Sequencing, 3D structure, Epitopes

## Abstract

**Background:**

HCV is causing hundreds of cases yearly in Pakistan and has become a threat for Pakistani population. HCV E2 protein is a transmembrane protein involved in viral attachment and thus can serve as an important target for vaccine development but because of its variability, vaccine development against it has become a challenge. Therefore, this study was designed to isolate the HCV E2 gene from Pakistani HCV infected patients of 3a genotype, to perform *In-silico* analysis of HCV E2 isolated in Pakistan and to analyze HCV E2 protein sequence in comparison with other E2 proteins belonging to 3a and 1a genotypes to find potential conserved B-cells and T-cell epitopes that can be important in designing novel inhibitory compounds and peptide vaccine against genotype 3a and 1a.

**Patients and methods:**

Patients were selected on the basis of elevated serum ALT and AST levels at least for six months, histological examination, and detection of serum HCV RNA anti-HCV antibodies (3^rd^ generation ELISA). RNA isolation, cDNA synthesis, amplification, cloning and sequencing was performed from 4 patient’s serum samples in order to get the HCV E2 sequence. HCV E2 protein of Pakistani origin was analyzed using various bioinformatics tools including sequence and structure tools.

**Results:**

HCV E1 protein modeling was performed with I-TASSER online server and quality of the model was assessed with ramchandran plot and Z-score. A total of 3 B-cell and 3 T-cell epitopes were found to be highly conserved among HCV 3a and 1a genotype.

**Conclusion:**

The present study revealed potential conserved B-cell and T-cell epitopes of the HCV E2 protein along with 3D protein modeling. These conserved B-cell and T-cell epitopes can be helpful in developing effective vaccines against HCV and thus limiting threats of HCV infection in Pakistan.

## Introduction

Hepatitis C virus (HCV) is a global health problem and a significant risk factor in developing liver associated diseases including hepatocellular carcinoma. HCV has affected 270 million people worldwide of which 10 million belongs to Pakistan [1]. Hundreds of HCV cases are reported each year in Pakistan and according to the prevalence analysis it is clear that HCV genotype 3a is most common in all provinces of Pakistan [2] except in Balochistan where the most prevalent subtype is 1a [3]. Due to six genotypes and their variability, HCV vaccine development has always been a challenge and for this, structural and non- structural proteins are being targeted to develop an effective vaccine.

HCV is a plus strand virus having a genomic RNA and viral envelope proteins, namely E1 and E2 [4] that are anchored in a host derived lipid protein membrane surrounding the nucleocapsid composed of several copies of core protein. E1 and E2 have molecular weights of 33–35 and 70–72 kDa, respectively [5-7]. E2 is highly glycosylated and contains up to 11 N-linked glycosylation sites, with most of the sites being well conserved. In addition, E2 contains hypervariable regions with amino acid sequences differing up to 80% between HCV genotypes and between subtypes of the same genotype [8-10]. E2 glycoprotein is a key molecule regulating the interaction of the HCV with cell surface proteins and binds to the major extracellular loop of human CD81, a tetraspanin expressed in various cell types including hepatocytes and B lymphocytes [11], its truncated forms also interacts with scavenger receptor type B class 1 protein (SRB-1) and high density lipoprotein (HDL) binding molecule [12-15]. Mannose binding proteins (DC-SIGN and L-SIGN) have been suggested to have interactions with the HCV E2 but their function in viral entry is unclear [16]. HCV E2 posses’ glycosylation sites which interact directly with cell surface receptors enabling the virus to enter the cell [17-20], therefore it is important to target this protein to stop viral entry.

For designing effective inhibitors against envelope proteins, it is important to have knowledge of sequence and structure of protein. Bioinformatics analysis has open new vistas to provide more insights into protein sequence and structural features. Therefore, this study was designed to isolate the HCV E2 sequence from HCV infected patients of 3a genotype and to analyze conservation and variability for designing conserved B-cells and T-cells epitopes. B-cell and T-cell epitopes are important in raising the desired immune responses and number of epitopes and modulation of immune recognition of antigens can be influenced by deglycosylation of viral glycoproteins [21]. As knowledge of epitopic regions on protein is important in designing effective inhibitors, [22] therefore, both B-cell and T-cell epitopes were predicted that were well conserved in the HCV E2 protein of genotype 3a and 1a.

## Methodology

### Source of serum samples

The local HCV 3a serum samples from 4 patients were collected from CAMB (Center for Applied Molecular Biology) diagnostic laboratory, Lahore, Pakistan after clinical diagnosis under the provision of the Institutional Review Board (IRB) of NCEMB (National Center of Excellence in Molecular Biology), University of the Punjab Lahore, Pakistan. The participating subjects gave informed consent for the collection of blood samples for this study. Patients were selected on the basis of elevated serum ALT and AST levels at least for six months, histological examination, and detection of serum HCV RNA anti-HCV antibodies (3^rd^ generation ELISA).

### RNA isolation, cDNA synthesis, amplification and cloning of the HCV E2 gene

All the steps of RNA isolation from serum samples were carried out in the type IIB Biosafety hood (Beckman Coulter, USA). RNA from collected serum samples was extracted using a PurescriptÂ® RNA Isolation kit (Gentra System Pennsylvania, USA) according to the manufacturer’s protocol. Extracted RNA was reverse transcribed into complementary DNA (cDNA) using Moloney murine leukemia virus reverse transcriptase (MMLV-RTase) (Fermentas, USA). A set of primers was designed for PCR amplification of the HCV E2 gene from cDNA of HCV 3a infected patients, against HCV isolate NZL1 (D17763) sequence retrieved from NCBI (National Center for Biotechnology Information) using Primer3 software (http://frodo.wi.mit.edu/) (Table 1). To efficiently produce the desired PCR products, the amplification was performed with 4 λ¼l of cDNA using forward and reverse primers in a thermal cycler with *Taq* DNA polymerase. PCR protocol was used that involved 35 cycling steps at 54Â°C annealing temperature. After the completion of PCR reactions, DNA was resolved on 1.2% TAE agarose gel along with 100 bp DNA size marker on the basis of molecular weight; mixing samples with 6x loading dye (Fermentas, USA). Then gel was observed under the ultra violet (U.V) light. Purification of DNA from the agarose gel slice was done with QIA quick gel extraction kit (Qiagen, USA). Individual PCR products were inserted into TA cloning vector, pCR2.1-TOPO (Invitrogen, USA). To confirm the HCV E2 insert in pCR2.1 vector, regular PCR was run as described above using gene specific primers and plasmid DNA as template. Moreover, restriction digestion of the pCR2.1 vector was done by endonuclease *EcoR1* restriction enzyme and incubated at 37Â°C for one hour with reaction mixture. All plasmid constructs were sequenced for confirmation of the insert at the standard cycling conditions. Sequence analysis of the plasmid DNA was performed according to the manufacturer’s protocol using BigDye™ Terminator v3.0 Cycle sequencing kit (Applied Biosystems, Germany). Sequencing for both positive and negative strands on an automated sequencer (Applied Biosystems 3700 DNA Analyzer, Germany) was performed. Three full length nucleotide sequences of HCV E2 nucleotide were submitted in the NCBI database having accession no. GQ355940, GQ355941 and GQ355942.

**Table 1:** Sequences of Primer used for PCR amplification of the HCV E2 gene

**No.**	**Primer name**	**Sequences 5λ„-3λ„**	**Primer size**	**PCR product size**
1	E2-F	CGCAATCATCATGGTTATGTTCTCA	25	1151 bp
2	E2-R	CCAAGGCTGCTTCTGTCTGTGATA	24	

### Sequence analysis, homology modeling and stereochemical analysis

HCV E2 sequence of 3a genotype (GQ355940.1) was used to develop three-dimensional structure of E2 protein through homology modeling because crystal or NMR structure of the HCV E2 protein was not available in Protein Data Bank (PDB) (http://www.rcsb.org/pdb/home/home.do). Different parameters of primary structure analysis were computed using ProtParam online tool [23]. The secondary structure of the protein was computed using different servers. DiANNA tool [24] was used to check the system classification and disulfide connectivity. This knowledge can be helpful in understanding the secondary structure of the protein since disulfide bond bridges are important in protein fold stabilization. The 3D model was generated using the I-TASSER online server [25] which generates 3D models along with their confidence score (C-Score). After generating 3D model, structure analysis and stereochemical analysis were performed using different evaluation and validation tools. The Psi/Phi Ramachandran plot was obtained using PROCHECK [26] which helped in evaluating backbone conformation. Ramachandran plot was also used to check non-GLY residues at the disallowed regions. Quality of the model was assured using Z - scores, which is indicative of overall model quality and to assure that the predicted structure is within range of score as found in native proteins. PROSA web tool [27] was used to determine Z-scores. Furthermore, the generated model was submitted in the Protein model database (PMDB) (http://mi.caspur.it/PMDB/main.php) having PMDB identifier PM0078776.

### T-cell epitope and B-cell epitope prediction

Transmembrane topology of the E2 protein was checked using TMHMM online tool [28] and antigenicity of protein was checked using Vexigen v2.0 online antigen prediction server [29]. T-cell epitopes were predicted using Epijen v1.0 [30] online server using HLA Alleles A*0101, A*0201, A*0202, A*0203, A*0206, A*0301, A*1101, B*07, B*51. Proteasome cutoff was set to a value of 0.1. TAP prediction cutoff was set to 5 and output cut off threshold was set to a 5%. Transmembrane localization of epitopes with minimum IC50 value was checked and epitopes that were present in transmembrane/exo-membrane region were selected and checked for potential antigen or not. Only epitopes that were in transmembrane/Exo-membrane region and have a potential antigenicity score were subjected to conservancy analysis. Furthermore, B-cell epitopes were predicted using BCPred [31] online server with 75% specificity criteria for epitope prediction. Epitopes exposed on the surface of the membrane were checked for their antigenecity using Vexijen v2. 0 online server. Both T-cell and B-cell epitopes were analyzed for their conservancy among all retrieved sequences of E2 belonging to genotype 3a and 1a. For this purpose, the IEDB Epitope conservancy analysis server [32] was utilized.

### Conservation of epitopes

The degree of conservation of amino acid depicts there structural and functional importance. For predicting effective and conserved peptides, E2 protein sequences belonging to HCV 3a and 1a genotype were retrieved from the NCBI protein database (Additional file 1) and were compared with the E2 sequence of Pakistan. Conservation and variation analysis of the HCV E2 was carried out through the IEDB conservancy analysis tool. As the HCV E2 protein is important in viral entry and highly variable, therefore it is important to identify conserved epitopes that can serve as best targets for potential inhibitors and vaccine.

## Results

**Figure 1:**
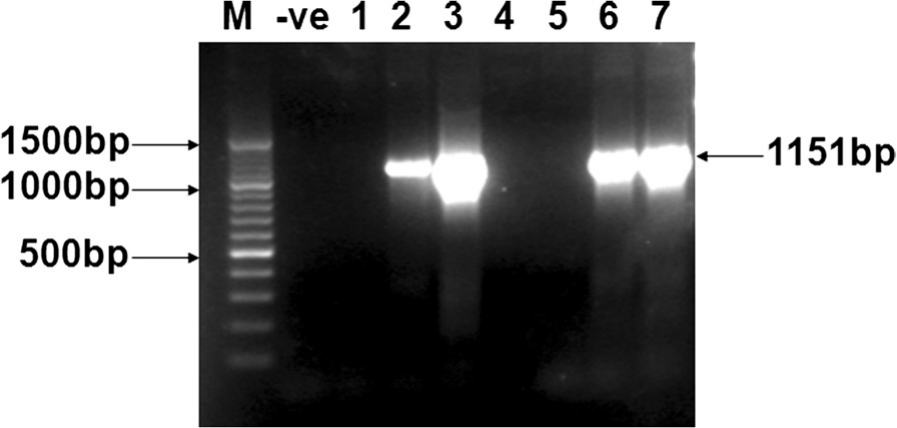
**HCV E2 gene amplification through PCR.** PCR amplification of E2 gene of HCV 3a genotype from different patients’ serum samples. Sample # 2, 3, 6, and 7 shows amplified PCR product of 1151 bp of E2. 100 bp DNA size Marker (M) and negative PCR (âˆ’ve) are shown. 1–7 samples are from different patients.

A total of 4 patients were selected for isolation and amplification of the HCV E2 sequence of 3a genotype. The template cDNA used for PCR amplification was obtained after the reverse transcription of RNA extracted from the serum of HCV patients. PCR was optimized and run at specific conditions of the primers to get a product of expected gene size. Figure 1 shows the PCR amplify fraction of the gene fragments i.e. 1151 bp of the E2 gene.

### Cloning and confirmation of HCV 3a E2 in pCR2.1 vector

**Figure 2:**
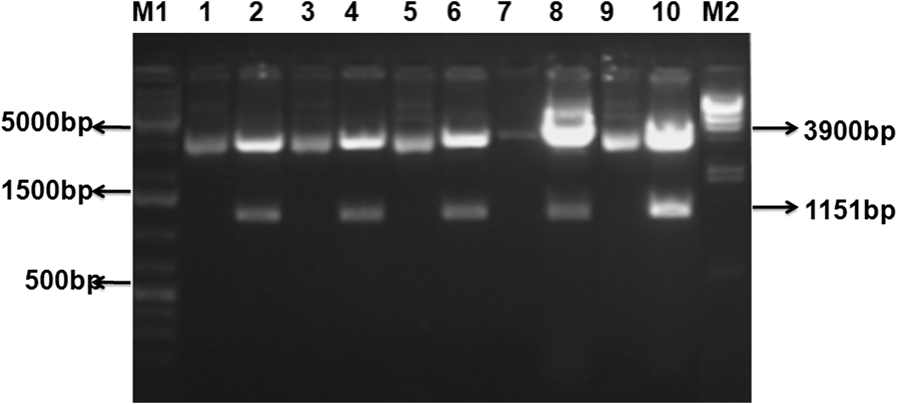
**Confirmation of expression vector via restriction digestion.** Restriction digestion of pCR2.1/E2 vector, sample #1-10 are digested (gene of 1151 bp) and undigested plasmid respectively. 1 Kb DNA Size Marker (M1) and λ/HindIII DNA size Marker (M2) are shown.

Gel purified PCR product of the HCV E2 gene was cloned into pCR2.1 TA cloning vector. PCR amplification was carried out for the confirmation of the HCV E2 gene cloning. Same PCR conditions were used for the amplification of the gene from the plasmid (pCR2.1/HCV E2 gene), the same size of PCR products was observed when run on 1.2% agarose gel. The PCR positive clones were used for further confirmation analysis by restriction digestion of the plasmid containing the HCV E2 (pCR2.1/HCV E2). Since pCR2.1 plasmid (3.9 KB) contained 2 *EcoRI* sites, just outside the cloning site in TA vector, restriction digestion with this enzyme result in the linear plasmid of 3.9 KB and the PCR fragment size of individual genes. Digested and undigested plasmids were run on 1% TAE agarose gel. Figure 2 shows the digested product of 3900 KB size for the plasmid and 1151 bp, fragments of HCV E2 when observed under the U.V light confirming the insertion of the HCV E2 gene.

### Structural description of the 3D model

**Figure 3:**
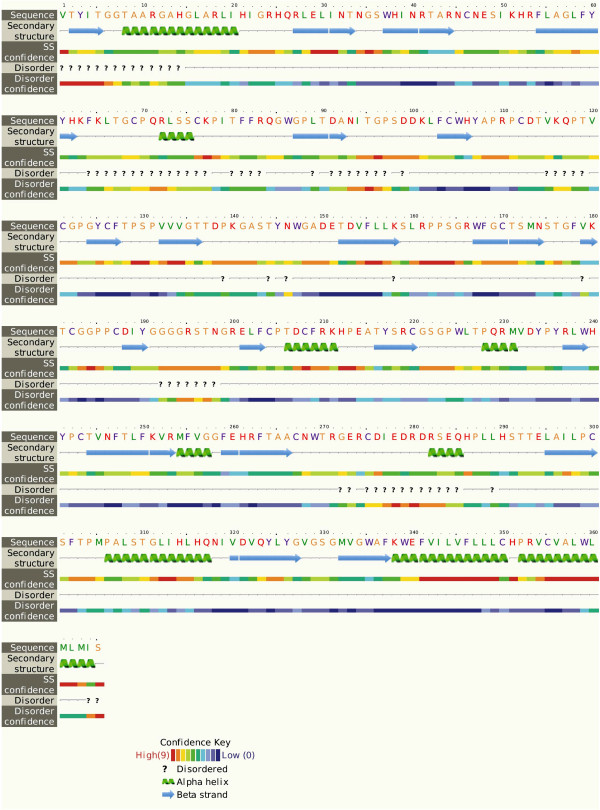
Secondary structure of HCV E2 of Pakistani origin.

**Figure 4:**
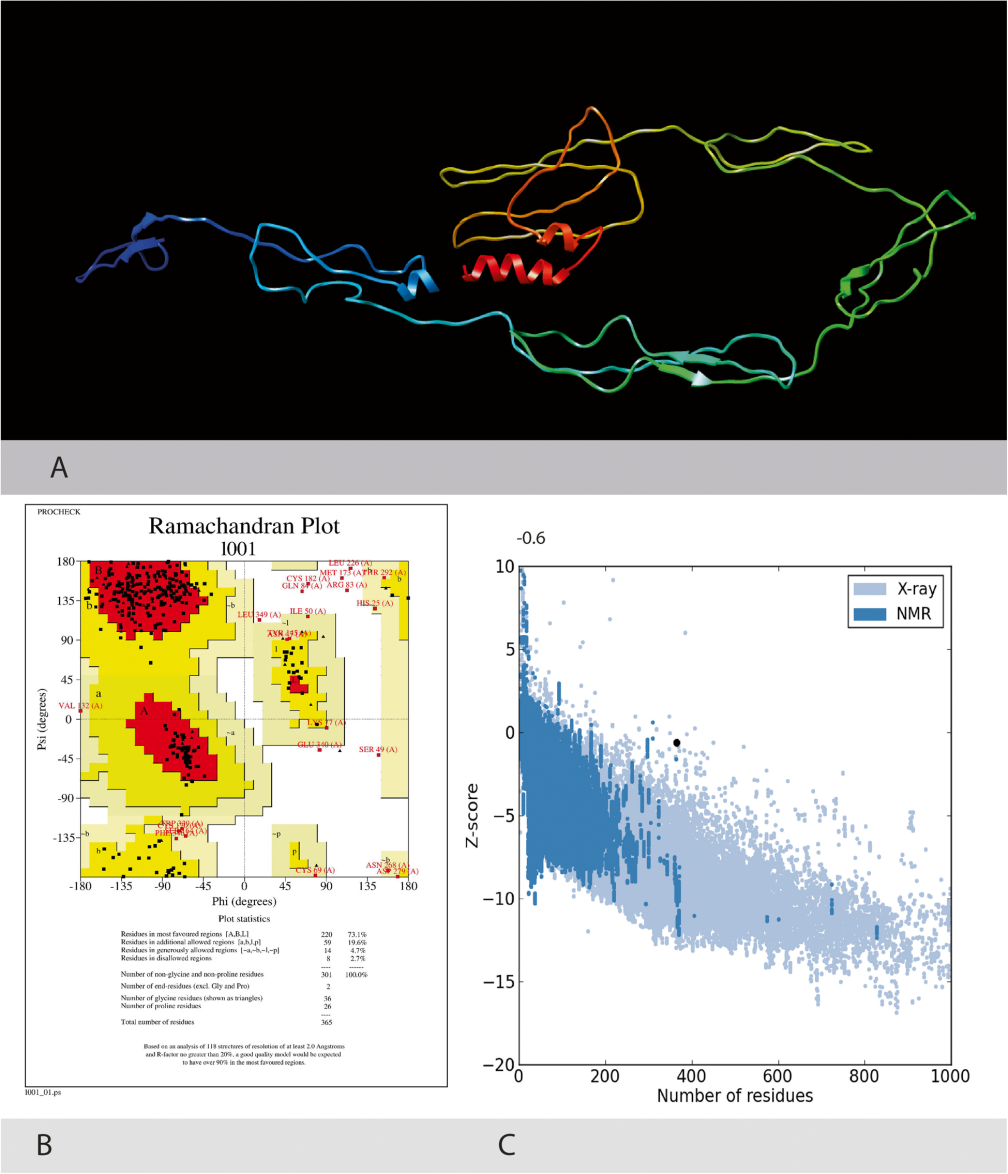
**3-D structure of the HCV E2 protein.A**. Predicted 3 Dimensional structure of the HCV Envelope protein 2 using Homology Modelling. **B**. Ramachandran plot showing residues in the most favorable region and disallowed regions. **C**. Z-score showing the quality of 3D structure.

The genetic diversity of the HCV largely impacts in the treatment management as well as the development of new HCV antiviral strategies. Sequence analysis of local HCV 3a E2 gene obtained after sequencing from different patients’ serum samples was performed using protparam, DiANNA, I-TASSER, Procheck and ProsA Z-Score. Physiochemical parameters of the HCV E2 protein showed that it is 365 aa long sequence and had a molecular weight of 41046.2 Daltons and theoretical isoelectric point (PI) of 8.95. An isoelectric point above 7 indicates a positively charged protein. The instability index (II) is computed to be 41.32. This classifies the protein as unstable. The N-terminus of the sequence is considered to be V (Val). The negative Grand average of hydropathicity (GRAVY) of âˆ’0.170 indicates that the protein is hydrophilic. Rich amounts of Glycine (G), Leucine (L), Threonine (T) and Proline (P) were found in the protein. Secondary structural features are shown in Figure 3. Disulfide bonds predicted by DiANNA are shown in the Table 2. Disulfide connectivity was predicted to be in between 1–8, 2–7, 3–12, 5–11, 6–10, 9–19, 13–17, 14–18, 15–16. Protein functions, interactions and localizations can be understood by the 3D structure of proteins [24], therefore, 3D structure of the HCV E2 protein was predicted using the I-TASSER online server and the best predicted structure with the maximum confidence score (C-Score âˆ’2.18) was selected (Figure 4A). Quality and reliability of the structure was checked using Z-score, and Ramachandram plot. The Stereochemical quality of 3D structure was checked by Ramachandran plot via analyzing residue-by-residue geometry and overall structure geometry. The result of the Ramachandran plot showed 73.1% of residues in the favorable region (Figure 4B). Overall model quality can be checked by ProsA Z-score, which is used to check whether the input structure is within the range of scores typically found for native proteins of similar size. The Z-score of the protein was âˆ’0.6 (Figure 4C). The Ramachandran plot and Z-score results confirmed the quality of the homology model of the HCV E2 protein.

**Table 2:** Predicted disulfide bonds

**Predicted bonds**	
**46 - 170**	RTARNCNESIK - GRWFGCTSMNS
**69 - 126**	FKLTGCPQRLS - CGPGYCFTPSP
**76 - 208**	QRLSSCKPITF - FCPTDCFRKHP
**112 - 204**	YAPRPCDTVKQ - GRELFCPTDCF
**121 - 187**	KQPTVCGPGYC - CGGPPCDIYGG
**182 - 355**	GFVKTCGGPPC - CHPRVCVALWL
**220 - 300**	ATYSRCGSGPW - LAILPCSFTPM
**243 - 350**	LWHYPCTVNFT - VFLLLCHPRVC
**267 - 275**	RFTAACNWTRG - TRGERCDIEDR

### Epitope prediction

The TMHMM online server showed that residues 1–340 presented outside region, residues 341–363 were within the transmembrane and residues 364–365 were inside the region of the protein. Vexijen v2. 0 showed an overall antigenic score of 0.4653.

### B-cell epitope prediction

B-cell epitopes are important for protection against virus infection. B-cell epitopes were predicted using BCPred having the criteria of length 9 and 75% specificity using BCPred algorithm. 19 epitopes were predicted and all of them were exposed outside of the membrane. Antigenecity of epitopes was checked using Vexijen v2.0 and it was found that out of 19 epitopes 7 were non-antigen thereby, resulting its exclusion (Table 3). Epitopes with antigenic properties can be important in raising the desired immune responses.

**Table 3:** B-cell epitopes with their antigenic score

**Position**	**Epitope**	**BCPred score**	**Antigenecity score**	**Antigen/non-antigen**
**136**	**TTDPKGAST**	**0.999**	**0.6265**	**Antigen**
278	EDRDRSEQH	0.999	âˆ’0.0020	Non-Antigen
**191**	**GGGGRSTNG**	**0.996**	**0.4167**	**Antigen**
**4**	**ITGGTAARG**	**0.994**	**2.7513**	**Antigen**
**268**	**NWTRGERCD**	**0.919**	**0.7977**	**Antigen**
92	ANITGPSDD	0.9	âˆ’0.3755	Non-Antigen
**316**	**HQNIVDVQY**	**0.892**	**1.5115**	**Antigen**
**115**	**VKQPTVCGP**	**0.891**	**0.5372**	**Antigen**
**303**	**TPMPALSTG**	**0.854**	**0.5271**	**Antigen**
**223**	**GPWLTPQRM**	**0.843**	**0.8162**	**Antigen**
**126**	**CFTPSPVVV**	**0.836**	**1.0952**	**Antigen**
169	GCTSMNSTG	0.821	0.3500	Non-Antigen
212	HPEATYSRC	0.777	âˆ’0.3926	Non-Antigen
**31**	**INTNGSWHI**	**0.766**	**1.0793**	**Antigen**
42	TARNCNESI	0.75	0.2912	Non-Antigen
**82**	**FRQGWGPLT**	**0.747**	**1.5574**	**Antigen**
180	KTCGGPPCD	0.725	âˆ’0.6164	Non-Antigen
**104**	**CWHYAPRPC**	**0.725**	**0.8901**	**Antigen**
65	KLTGCPQRL	0.723	âˆ’0.4238	Non-Antigen

Non-antigens are shown in bold text.

### T-cell epitope prediction

Epijen online server predicted the T-cell epitopes on the basis of the IC50 value. Epitopes that had minimum IC50 value and exposed outside of the membrane were checked for their antigenecity using Vexijen v2. 0 (Table 4). Epitopes at position 86, 132, 237, 307, 314, and 335 were found to be the probable antigen and were used for conservation analysis.

**Table 4:** T-cell epitopes on the basis of minimum IC50 value and antigenic score

**Position**	**Epitope**	**Predicted IC**_**50 **_**value (nM)**	**Allele**	**Antigenic score**	**Antigen/non-antigen**
226	LTPQRMVDY	7.57	A*0101	0.3723	Non-Antigen
**237**	**RLWHYPCTV**	**0.12**	A*0201	**0.4100**	**Antigen**
346	FLLLCHPRV	0.17	A*0201	0.0731	Non-Antigen
324	YLYGVGSGM	0.24	A*0201	0.3163	Non-Antigen
**307**	**ALSTGLIHL**	**0.43**	A*0201	**0.9005**	**Antigen**
248	TLFKVRMFV	0.86	A*0202	âˆ’0.1899	Non-Antigen
**307**	**ALSTGLIHL**	**2.25**	A*0202	**0.9005**	**Antigen**
65	KLTGCPQRL	3.09	A*0202	âˆ’0.4238	Non-Antigen
**237**	**RLWHYPCTV**	**3.46**	A*0202	**0.4100**	**Antigen**
**307**	**ALSTGLIHL**	**0.10**	A*0203	**0.9005**	**Antigen**
65	KLTGCPQRL	0.17	A*0203	âˆ’0.4238	Non-Antigen
311	GLIHLHQNI	0.17	A*0203	0.2190	Non-Antigen
**314**	**HLHQNIVDV**	**1.19**	A*0203	**0.5275**	**Antigen**
312	LIHLHQNIV	3.80	A*0206	0.0960	Non-Antigen
248	TLFKVRMFV	7.45	A*0206	âˆ’0.1899	Non-Antigen
57	GLFYYHKFK	0.25	A*0301	âˆ’0.0505	Non-Antigen
93	NITGPSDDK	0.47	A*0301	0.0978	Non-Antigen
243	CTVNFTLFK	3.16	A*0301	0.1623	Non-Antigen
**132**	**VVVGTTDPK**	**2.88**	A*1101	**1.8674**	**Antigen**
172	SMNSTGFVK	3.30	A*1101	0.3124	Non-Antigen
305	MPALSTGLI	4.62	B*07	0.3991	Non-Antigen
355	CVALWLMLM	6.58	B*07	âˆ’0.0937	Non-Antigen
**335**	**WAFKWEFVI**	**7.59**	B*51	**1.3516**	**Antigen**
**86**	**WGPLTDANI**	**8.69**	B*51	**0.8344**	**Antigen**

Non-Antigens are shown in bold text.

### Conservation of epitopes

A total of 44 sequences of the HCV E2 of 1a and a total of 50 sequences of 3a was retrieved from the NCBI protein database. The IEDB conservancy analysis tool was used to check the conservancy of antigneically effective epitopes (B-cell and T-cell). B-Cell epitope NWTRGERCD at position 268, HQNIVDVQY at position 316 and CFTPSPVVV at position 126 were found to be conserved. T-Cell epitope RLWHYPCTV at position 237 for HLA: A*0201, ALSTGLIHL at position 307 for HLA: A*0201, A*0202 and A*0203 and HLHQNIVDV at position 314 for HLA: A*0203 were also found to be conserved. Conserved B-Cell and T-Cell epitopes are shown in the Table 5. Epitope HQNIVDVQY at position 316 had a maximum antigenic score (1.5115) ensuring maximum bonding.

**Table 5:** Conserved B-cell and T-cell epitopes

**Epitope**	**B-cell/T-cell**
NWTRGERCD	B-Cell
HQNIVDVQY	B-Cell
CFTPSPVVV	B-Cell
RLWHYPCTV	T-Cell (A*0201, A*0202)
ALSTGLIHL	T-Cell (A*0201, A*0202, A*0203)
HLHQNIVDV	T-Cell (A*0203)

## Discussion

Advancements in biotechnology and knowledge of immune responses have opened new doors for vaccine development and implementation. Discovery of vaccine using genetic information through *in-silico* approach rather than in-vitro study is called as reverse vaccinology [33]. Reverse Vaccinology takes advantage of the genome sequence of the pathogen. This approach helps in identifying all antigens of pathogens and also allows the discovery of novel antigens [34]. Gene sequences of viral pathogens have been used to develop synthetic peptides, used for vaccines against chronic infections such as Hepatitis B, Hepatitis C and HIV. Peptide based vaccines have shown efficacy in clinical trials and this efficacy correlate with the induction of the T cell-specific immunity. Bioinformatics resources, store and organize immune reactivity and pathogen data and availability of genomic sequences of pathogens can provide new information on cancer-specific epitopes and increase our knowledge to design novel peptide vaccines [35]. Many vaccines that were impossible to develop have now become a reality [34]. The most common HCV genotype in Pakistan is 3a while 1a is common in Balochistan with a strong correlation between chronic HCV infection (genotype 3a) and HCC in Pakistan [2].

This study was designed to perform *in-silico* analysis of the HCV E2 protein isolated in Pakistan. For this purpose, different sequence and structure analysis tools were used to explore the insights of the HCV E2 and to compare the HCV E2 sequence of Pakistan with other Pakistani E2 sequences. There is currently no high-resolution structure of the HCV E2 glycoprotein to further understand its mechanism of viral entry or immune evasion [36]. We used a homology modeling approach to predict the 3D structure of the HCV E2 protein of Pakistan. The predicted 3D structure will provide more insight in understanding the structure and function of the protein. Moreover, this structure can be used for drug designing or understanding the interactions between proteins. As a part of the present study, we predicted conserved T-cell and B-cell epitopes that can be used as the target for vaccine development against HCV genotype 3a and 1a. Among all the predicted B-cell epitopes, 12 epitopes were found to be antigenically effective and all these epitopes were in the exo-membrane region of the protein. After conservation analysis it was found that only 3 epitopes were conserved with other E2 sequences of genotype 3a and 1a. For T-cell epitope mapping, the Epijen online server was used. A total of 25 epitopes with minimum IC50 value were selected and 9 were found to be antigenically effective, but only 3 T-cell epitopes were found to be well conserved in E2 sequences of genotype 3a and 1a.

## Conclusion

Multiple antigenic components of the virus can be a important target to develop effective vaccines, thus directing the immune system to protect the host from the virus. In Pakistan, genotype 3a is the most prevalent genotype followed by 3b and 1a. Keeping this in mind, this study was conducted to perform sequence, structure, and conservation/variation analysis along with homology modeling of the HCV E2 protein of Pakistani origin. This study revealed B-cell and T-cell epitopes that are conserved in 3a and 1a E2 protein of HCV. For diagnosing HCV genotype 3a and 1a, these conserved epitopes may be highly useful and may also help in developing a successful vaccine that can target both 3a and 1a genotypes.

## Competing interests

The authors declare that they have no competing interests.

## Authors’ contribution

UAA designed the study, and SI wrote the manuscript. SK performed cloning work, UAA and SI performed all in-silico work, and UAA critically reviewed the manuscript. All authors read and approved the final manuscript.

## Authors’ information

Sobia Idrees (MPhil student), Usman A Ashfaq (PhD molecular Biolog), Saba Khaliq (PhD molecular biology).

## Supplementary Material

Additional file 1 Genotype 1a sequences. Click here for file
