# Supplementary material for: HCV Envelope protein 2 sequence comparison of Pakistani isolate and In-silico prediction of conserved epitopes for vaccine development
Source: J Transl Med. 2013 Apr 30;11:105. doi: 10.1186/1479-5876-11-105 (PMC3663723; doi:10.1186/1479-5876-11-105)
Supplement: Additional file 1 — Genotype 1a sequences. [file 1479-5876-11-105-S1.doc]

**Genotype 1a Sequences:**

>gi|291501301|gb|ADE08458.1| polyprotein [Hepatitis C virus subtype 1a]

ETHVTGGSAGRAASGVAGLFSPGPRQNIQLINTNGSWHINRTALNCNGSLDAGWIVGLFYYHKFNSSGCP

ERLASCRPLADFDQGWGPISYTNGSGPEHRPYCWHYPPKPCGIVPARNVCGPVYCFTPSPVVVGTTDRAG

VPTYNWGGNETDVFVLNNTRPPLGNWFGATWMNSSGYTKVCGAPPCVIGGVGNNTLHCPTDCFRKHPEAT

YSRCGSGPWITPRCLVHYPYRLWHYPCTINYTLFKVRMYVGGVEHRLEAACNWTRGERCDLDDRDRSELS

PLLLSTTQWQVLPCSFTTLPALTTGLIHLHQNIVDVQYLYGVGSSVVSWAIQWEYIVLLFLLLADARVCS

CLWMMLLISQAEA

>gi|309252623|gb|ADO60285.1| polyprotein [Hepatitis C virus subtype 1a]

ETHVTGGTAARPASGVAGLFSRGPRLNIQLINSNGNWHINRTALTCNGSLDAGWIVGLFYYNKFNSSGFP

ERLASCRPLADFDQGWGPISYTNGSGPEHRPYCWHYPPKPCAIVPARNVCGPVYWFTPSPVVVGTNDRAG

VPTYNWGGNETDVFVLNNTRPPLGNWFGATWMNSSGYTKVCGAPLCVIGGVANNTLHCPTDCFRKHPEAT

YSRCGSGPWITPRCLVHYPYRLWHYPCTINYTLFMVRMYVGGFEHRLEAACNWTRGERCDLDDRDRSELS

PLLLSTTQWQVLPCSFTTLPALTTGLIHLHQNIVDVQYLYGVGSSVVSWAIQWEYIVLLFLLLADARVCS

CLWMMLLISQAEA

>gi|309252621|gb|ADO60284.1| polyprotein [Hepatitis C virus subtype 1a]

ETHVTGGTAGRAASGVAGLFSRGPRQNIQLINSNGNWHINRTALTCNGSLDAGWIVGLFYYHKFNSSGFP

ERLASCRPLADFDQGWGPISYTNGSGPEHRPYCWHYPPKPCGIVPARNVCGPVYWFTPSPVVVGTTDRAG

VPTYNWGGNETDVFVLNNTRPPLGNWFGATWMNSSGYTKVCGAPPCVIGGVANNTLHCPTDCFRKHPEAT

YSRCGSGPWITPRCLVHYPYRLWHYPCTINYTLFMVRMYVGGFEHRLEAACNWTRGERCDLDDRDRSELS

PLLLSTTQWQVLPCSFTTLPALTTGLIHLHQNIVDVQYLYGVGSSVVSWAIQWEYIVLLFLLLADARVCS

CLWMMLLISQAEA

>gi|341940014|gb|AEL12151.1| polyprotein [Hepatitis C virus]

MSTNPKPQRKTKRNTNRRPQDVKFPGGGQIVGGVYLLPRRGPRLGVRATRKTSERSQPRGRRQPIPKARQ

PEGRTWAQPGYPWPLYGNEGCGWAGWLLSPRGSRPSWGPTDPRRRSRNLGKVIDTLTCGFADLMGYIPLV

GAPLGGAARALAHGVRVLEDGVNYATGNLPGCSFSIFLLALLSCLTVPASAWPTKCAIPRGFTMSPRKPQ

LEYCVRDGRYHPTLSRVCPLRSRGQRLEMLGADSPHSRHQGRQPPHNAASTPYRSACRGRHPLLGPLCGG

PVRVHLPCRSTVHFLPRPPLDNAKLQLFYVPGHITGHRMAWDMMMTGPLRRRWWLLSYSGPTSHLGHDRW

GPLGSPSGHSVFFHVGELGEGPDCVVTIAGVDASTTITGGSVAQDPSGLSKFFSSGRKQDIQLINSNGSW

HINRTALNCNASLDAGWMAGLFYYHKFNSSGCPERMASCRPLADFAQGWGPISYANGSGPEHRPYCWHYP

PKPCGIVPARNVCGPVYLFPPSPVVVGTTGKVGLAYFQLGYQYHGRFFSNQHQAPVGQIVGLHLDEIFRI

HQSVRGASLFPPRGGQQHLAMPHDLFPQASRKHILSVWVRSLDHAQESGPLLLRVLALSLHRQLHPVQSQ

DVCGRGRAQAGSCLQLDAGRACRSGRQGQVRAQQVAAVHHTLSGSPCSFTTLPALTTGLIHLHQNIVDVQ

YLYGVGSSIVSWAIKWEYVILLFLLLADARICACLWMMLLISQAEA

>gi|339267825|gb|AEJ36324.1| polyprotein [Hepatitis C virus]

CFTPSPVVVGTTDRAGVPTHNWGENETDVFVLNNTRPPLGNWFGCTWMNSSGYTKVCGAPPCVIGGVGNN

TLHCPTDCFRKHPEATYSRCGSGPWITPRRLVHYPYRLWHYPCTINYTLFKVRMYVGGVEHRLEAACNWT

RGERCDLDDRDRSELSPLLLSTTQWQVLPCSFTTLPALTTGLIHLHQNIVDVQYLYGVGS

>gi|262385651|gb|ACY64765.1| envelope protein 2 [Hepatitis C virus subtype 1a]

ETHTTGGSVAHGASIIAGLFNQGSQQNIQLINTNGSWHINRTALNCNDSLSTGWIAGLLYRNKFNSSGCP

ERMASCKPLADFDQGWGPISYANGSGPDHRPYCWHYPPKPCGIVPXXNVCGPVYCFTPSPVVVGTTDRAG

VPTLNWGSNETDVFVLNNTRPPLGNWFGCTWMNSSGYTKVCGAPPCVIGGVGNNTLHCPTDCFRKHPDAT

YSRCGSGPWITPRCLVHYAYRLWHYPCTINYTLFKVRMYVGGVEHRLDVACNWTRGERCNLDDRDRSELS

PLLLXTTQWQVLPCSFTTLPALTTGLIHLHQNIVDVQYLYGVGSSIVSWAIKWEYVVLLFL

>gi|262385647|gb|ACY64763.1| envelope protein 2 [Hepatitis C virus subtype 1a]

QTRVTGGNAAANAYGLASLFSTGAKQNIQLINSNGSWHINRTALNCNASLDTGWVAGLFYYHKFNSSGCP

ERMASCRPLADFDQGWGPISYANGSGPEHRPYCWHYPPKPCGIVPAQNVCGPVYCFTPSPVVVGTTDKLG

APTYNWGSNDTDVLVLNNTRPPLGNWFGCTWMNSSGFTKVCGAPPCXIGGGGNNTLYCPTDCFRKHPEAT

YSRCGSGPWLTPRCLVDYPYRLWHYPCTVNYTLFKVRMYVGGVEHRLGVACNWTRGERCBLDDRDRSELS

PLLLSTTQWQVLPCSFTTLPALTTGLIHLHQNIVDVQYLYGVGSSIVSWAIKWEYVILLFLLLADARICS

CLWMMLLI

>gi|262385649|gb|ACY64764.1| envelope protein 2 [Hepatitis C virus subtype 1a]

TTHVTGGTAGRATYGIARLFTPGAKQNIQLVNTNGSWHINRTALNCNASLDTGWVAGLFYYHQFNSTGCA

ARMASCKLLADFDQGWGPISHANGSGPETRPYCWHYPPKPCGIVPAQSVCGPVYCFTPSPVVVGTTDRLG

APTYNWGSNDTDVLVLNNTRPPLGNWFGCTWMNSSGFTKVCGAPPCVIGGAGNNTLHCPTDCFRKHPEAT

YSRCGSGPWLTPRCLVHYPYRLWHYPCTVNYTLFKVRMYVGGVEHRLEVACNWTRGERCDLDDRDRSELS

PLLLSTTQWQVLPCSFTTLPALTTGLIHLHQNIVDIQYLYGVGSSIVSWAIKWEYVVLLFLLLADARICS

CLWMMXLI

>gi|262385645|gb|ACY64762.1| envelope protein 2 [Hepatitis C virus subtype 1a]

TTYTXGGAAAQTTSFLAGFFKPGAKQDIQLINTNGSWHINRTALNCNASLETGWVAGLIYYHKFNSTGCR

ERMASCRPLADFDQGWGPISYANGSGPEHRPYCWHYPPKPCGIVPAXNVCGPVYCFTPSPVVVGTTDKLG

APTYNWGGNDTDVFVLNNTRPPLGNWFGCTWMNSSGFTKVCGAPPCVIGGVGNNTLHCPTDCFRKHPEAT

YSRCGSGPWITPRCLVHYAYRLWHYPCTLNYTLFKVRMYVGGVEHRLEVACNWTRGERCDLDDRDRSELS

PLLLSTTQWQVLPCSFTTLPALTTGLIHLHQNIVDVQYLYGVGSSIVSWAIKWEYVVLLFLLLADARICS

CLWMMLLI

>gi|262387382|gb|ACY65429.1| envelope protein 2 [Hepatitis C virus subtype 1a]

MMMNWSPTTALVVAQLLRIPQAILDMIAGAHWGVLAGIAYFSMVGNWAKVVVMLLLFAGVDAETHTTGAV

AGRVTSSFTSFFMQGPKQNIQLINSNGSWHINRTALNCNDSLSTGWVAGLFYYSKFNSSGCPERMASCRP

LADFAQGWGPIRYANGSGPDHRPYCWHYPPKPCGIVPARSVCGPVYCFTPSPVAVGTTDRSGVPTYNWGG

NETGVFILNNTRPPQGNWFGCTWMNSTGYTKVCGAPLCDIGGVGNNTLRCPTDCFRKHPEATYSRCGSGP

WLTPRCLVDYPYRLWHYPCTVNYSIFKVRMYVGGVEHRLEAACNWTRGERCDLEDRDRSELSPLLLSTTQ

WQVLPCSFTALPALSTGLIHLHQNIVDVQYLYGVGSSIASWAIKWEYVVLLFLLLADARVCSCLWMMLLI

>gi|262387378|gb|ACY65427.1| envelope protein 2 [Hepatitis C virus subtype 1a]

MMMNWSLTTALVVVQLPGIPQAILDMIAGAHWGVLAGIAYFSMVGNWAKVVVVLLLFAGVDAETHTTGAV

AGRVTSSFTSFFMQGPKQNIQLINPNGSWHINRAALNCNDSLSTGWVAGLLYYSKFNSSGCPERMASCRP

LADFAQGWGPIRYANGSGPDHRPYCWHYPPKPCGIVPARSVCGPVYCFTPSPVAVGTTDGSGVPTYSWGE

NETDVSILNNTRPPQGNWFGCTWMNSTGYTKVCGAPPCDIGGVGNNTLRCPTDCFRKHPEATYSRCGSGP

WLTPRCLVDYPYRLWHYPCTVNYSIFKVRMYVGGVEHRLEAACNWTRGERCDLEDRDRSELSPLLLSTTQ

WQVLPCSFTTLPALSTGLIHLHQNIVDVQYLYGVGSSIASWAIKWEYVVLLFLLLADARVCSCLWMMLLI

>gi|262387374|gb|ACY65425.1| envelope protein 2 [Hepatitis C virus subtype 1a]

MMMNWSPTTALVVVQLLRIPQAILDMIAGAHWGVLAGIAYFSMVGNWAKVVVVLLLFAGVDAETHTTGAV

AGHFTRSLTSVFTQGAKQNIQLINSNGSWHINRTALNCNDSLSTGWLVGLLYYSKFNSSGCPERMASCRP

LADFAQGWGPIRYANGSGPDHRPYCWHYPPKPCGTVPARSVCGPVYCFTPSPVAVGTTDRSGVPTYNWGG

NETDVFILNNTRPPQGNWFGCTWMNSTGYTKVCGAPPCDIGGVGNNTLRCPTDCFRKHPEATYSRCGSGP

WLTPRCLVDYPYRLWHYPCTVNYSIFKVRMYVGGVEHRLEAACNWTRGERCDLEDRDRSELSPLLLSTTQ

WQVLPCSFTTLPALSTGLIHLHQNIVDVQYLYGVGSSIASWAIKWEYVVLLFLLLADARVCSCLWMMLLI

>gi|262387370|gb|ACY65423.1| envelope protein 2 [Hepatitis C virus subtype 1a]

MMINWSPTTALVVAQLLRIPQAILDMIAGAHWGVLAGIAYFSMVGNWAKVVVVLLLFAGVDAETNTTGAV

AGRVTSSFTSFFMQGPKQNIQLTNSNGSWHINRTALNCNDSLSTGWVAGLLYYSKFNSSGCPERMASCRP

LADFAQGWGPIRYANGSGPDHRPYCWHYPPKPCGIVPARSVCGPVYCFTPSPVAVGTTDRSGVPTYNWGG

NETDVFILNNTRPPQGNWFGCTWMNSTGYTKVCGAPPCDIGGVGNNTLRCPTDCFRKHPEATYSRCGSGP

WLTPRCLVDYPYRLWHYPCTVNYSIFKVRMYVGGVEHRLEAACNWTRGERCDLEDRDRSELSPLLLSTTQ

WQVLPCSFTTLPALSTGLIHLHQNIVDVQYLYGVGSSIASWAIKWEYVVLLFLLLADARVCSCLWMMLLI

>gi|262387366|gb|ACY65421.1| envelope protein 2 [Hepatitis C virus subtype 1a]

MMMNWFPTMELVVDQFVRIPQAILDMIAGAHWGVLAGIEYFSMVGNWAKVVVVLLLFAGVDAETHTTGAV

AGHFTRSLTSVFTQGAKQNIQLINSNGSWHINRTALNCNDSLSTGWLAGLFYYSKFNSSGCPERMASCRP

LADFAQGWGPIRYANGSGPDHRPYCWHYPPKPCGIVPARSVCGPVYCFTPSPVAVGTTDRSGVPTYNWGG

NETDVFILNNTRPPQGNWFGCTWMNSTGYTKVCGAPPCDIGGVGNNTLRCPTDCFRKHPEATYSRCGSGP

WLTPRCLVDYPYRLWHYPCTVNYSILKVRMYVGGVEHRLEAACNWTRGERCDLEDRDRSELSPLLPSTTQ

WQVLPCSFTTLPALSTGLIHLHQNIVDVQYLYGVGSSIASWAIKWEYVVLLFLLLADARVCSCLWMMLLV

>gi|262387362|gb|ACY65419.1| envelope protein 2 [Hepatitis C virus subtype 1a]

MMMNWSPTTALVVAQLLRIPQAILDMIAGAHWGVLAGIAYFSMVGNWAKVVVVLLLFAGVDAETHTTGAV

AGRVTSSFTSFFMQGPKQNIQLINSNGSWHINRTALNCNDSLSTGWVAGLLYYSKFNSSGCPERMASCRP

LADFAQGWGPIRYANGSGPDHRPYCWHYPPKPCGIVPARSVCGPVYCFTPSPVAVGTTDRSGVPTYNWGG

NETDVFILNNTRPPQGNWFGCTWMNSTGYTKVCGAPPCDIGGVGNNTLRCPTDCFRKHPEATYSRCGSGP

WLTPRCLVDYPYRLWHYPCTVNYSIFKVRMYVGGVEHRLEAACNWTRGERCDLEDRDRSELSPLLLSTTQ

WQVLPCSFTTLPALSTGLIHLHQNIVDVQYLYGVGSSIASWAIKWEYVVLLFLLLADARVCSCLWMMLLI

>gi|262387358|gb|ACY65417.1| envelope protein 2 [Hepatitis C virus subtype 1a]

MMMNWSPTTALVVAQLLRIPQAILDMIAGAHWGVLAGIAYFSMVGNWAKVVVVLLLFAGVDAETHTTGAV

AGRVTSSFTSFFMQGPKQNIQLINSNGSWHINRTALNCNDSLSTGWVAGLLYYSKFNSSGCPERMASCRP

LADFAQGWGPIRYANGSGPDHRPYCWHYPPRPCGIVPARSVCGPVYCFTPSPVAVGTTDGSGVPTYSWGE

NETDVFILNNTRPPQGNWFGCTWMNSTGYTKVCGAPPCDIGGVGNNTLRCPTDCFRKHPEATYSRCGSGP

WLTPRCLVDYPYRLWHYPCTVNYSIFKVRMYVGGVEHRLEAACNWTRGERCDLEDRDRSELSPLLLSTTQ

WQVLPCSFTTLPALSTGLIHLHQNIVDVQYLYGVGSSIASWAIKWEYVVLLFLLLADARVCSCLWMMLLI

>gi|262387354|gb|ACY65415.1| envelope protein 2 [Hepatitis C virus subtype 1a]

MMMNWSPTTAFVVAQLLRIPQAILDMIAGAHWGVLAGIAYFSMVGNWAKVVVVLLLFAGVDAETHTTGAV

AGRVTSSFTSFFMQGPKQNIQLINSNGSWHINRTALNCNDSLSTGWVAGLFYYSKFNSSGCPERMASCRP

LADFAQGWGPIRYANGSGPDHRPYCWHYPPKPCGIVPARSVCGPVYCFTPSPVAVGTTDRSGVPTYNWGG

NETDVFILNNTRPPQGNWFGCTWMNSTGYTKVCGAPPCDIGGVGNNTLRCPTDCFRKHPEATYSRCGSGP

WLTPRCLVDYPYRLWHYPCTVNYSIFKVRMYVGGVEHRLEAACNWTRGERCDLEDRDRSELSPLLLSTTQ

WQVLPCSFTTLPALSTGLIHLHQNIVDVQYLYGVGSSIASWAIKWEYVVLLFLLLADARVCSCLWMMLLI

>gi|262387350|gb|ACY65413.1| envelope protein 2 [Hepatitis C virus subtype 1a]

MMMNWSPTTALVVAQLLRIPQAILDMIAGAHWGVLAGIAYFSMVGNWAKVVVVLLLFAGVDAETHTTGAV

AGRVTSSFTSFFMQGPKQNIQLINSNGSWHINRTALNCNDSLSTGWVAGLFYYSKFNSSGCPERMASCRP

LADFAQGWGPIRYANGSGPDHRPYCWHYPPKPCGIVPARSVCGPVYCFTPSPVAVGTTDRSGVPTYNWGG

NETDVFILNNTRPPQGNWFGCTWMNSTGYTKVCGAPPCDIGGVGNNTLRCPTDCFRKHPEATYSRCGSGP

WLTPRCLVDYPYRLWHYPCTVNYSIFKVRMYVGGVEHRLEAACNWTRGERCDLEDRDRSELSPLLLSTTQ

WQVLPCSFTTLPALSTGLIHLHQNIVDVQYLYGVGSSIAFWAIKWEYVVLLFLLLADARVCSCLWMMLLI

>gi|262387346|gb|ACY65411.1| envelope protein 2 [Hepatitis C virus subtype 1a]

MMMNWSPTTALVVDQLLRIPQAILDMIAGAHWGVLAGIAYFSMVGNWAKVVVVLLLFAGVDAETHTTGAV

AGRVTSSFTSFFMQGPKQNIQLINSNGSWHINRTALNCNDSLSTGWVAGLLYYSKFNSSGCPERMASCRP

LADFAQGWGPIRYANGSGPDHRPYCWHYPPKPCGIVPARSVCGPVYCFTPSPVAVGTTDRSGVPTYNWGG

NETDVFILNNTRPPQGNWFGCTWMNSTGYTKVCGAPPCDIGGVGNNTLRCPTDCFRKHPEATYSRCGSGP

WLTPRCLVDYPYRLWHYPCTVNYSIFKVRMYVGGVEHRLEAACNWTRGERCDLEDRDRSELSPLLLSTTQ

WQVLPCSFTTLPALSTGLIHLHQNIVDVQYLYGVGSSIASWAIKWEYVVLLFLLLADARVCSCLWMMLLV

>gi|262387266|gb|ACY65371.1| envelope protein 2 [Hepatitis C virus subtype 1a]

MMMNWSPTTALVGAQLLRIPQAILDMIAGAHWAVLGGIAYFSMVGNWAKVLVVLLLFAGVDAETHVTGGS

AGHAAFGIANLFRSGPQQNIQLVNTNGSWHINRTALNCNDSLNTGWIAGLLYYNRFNSSGCTERLASCRP

LTSFAQGWGPISYTNGTGPDQRPYCWHYPPKPCGIVPAKSVCGPVYCFTPSPVVVGTTDRSGAPTYNWGD

NDTDVLVLNNTRPPLGNWFGCTWMNSTGFTKVCGAPPCAIGGVGNHTLLCPTDCFRKHPEATYSRCGSGP

WLTPRCLVNYPYRLWHYPCTINYTIFKVRMYVGGVEHRLEAACNWTRGERCNPEDRDRSELSPLLLSTTQ

WQVLPCSFTTLPALSTGLIHLHQNIVDVQYLYGVGSSIASWAIKWEYVVLLFLLLADARVCSCLWMMLLI

>gi|262387262|gb|ACY65369.1| envelope protein 2 [Hepatitis C virus subtype 1a]

MMMNWSLTTALVVVQLLRIPQAILDMIAGAHWGVLAGIAYFSMVGNWAKVLVVLLLFAGVDAETHVTGGS

AGHAAFGIANLFRSGPQQNIQLVNTNGSWHINRTALNCNDSLNTGWIAGLLYYNRFNSSGCTERLASCRP

LTSFAQGWGPISYTNGTGPDQRPYCWHYPPKPCGIVPAKSVCGPVYCFTPSPVVVGTTDRSGAPTYNWGD

NDTDVLVLNNTRPPLGNWFGCTWMNSTGFTKVCGAPPCAIGGVGNHTLLCPTDCFRKHPEATYSRCGSGP

WLTPRCLVNYPYRLWHYPCTINYTIFRVRMYVGGVEHRLEAACNWTRGERCNLEDRDRSEFSPLLLSTTQ

WQVLPCSFTTLPALSTGLIHLHQNIVDVQYLYGVGSSIASWAIKWEYVVLLFLLLADARVCSCLWMMLLV

>gi|262387258|gb|ACY65367.1| envelope protein 2 [Hepatitis C virus subtype 1a]

MMMNWSPTTALVVAQLLRIPQAILDMIAGAHWGVLAGIAYFSMVGNWAKVLVVLLLFAGVDAETHVTGGS

AGHAAFGIANLFRSGPQQNIQLVNTNGSWHINRTALNCNDSLNTGWIAGLLYYNRFNSSGCTERLASCRP

LTSFAQGWGPISYTNGTGPDQRPYCWHYPPKPCGIVPAKSVCGPVYCFTPSPVVVGTTDRSGAPTYNWGD

NDTDVLVLNNTRPPLGNWFGCTWMNSTGFTKVCGAPPCAIGGVGNHTLLCPTDCFRKHPEATYSRCGSGP

WLTPRRLVNYPYRLWHYPCTINYTIFKVRMYVGGVEHRLEAACNWTRGERCNLEDRDRSELSPLLLSTTQ

WQVLPCSFTTLPALSTGLIHLHQNIVDVQYLYGVGSSIASWAIKWEYVVLLFLLLADARVCSCLWMMLLI

>gi|262387254|gb|ACY65365.1| envelope protein 2 [Hepatitis C virus subtype 1a]

MMMNWSPTTALVVAQLLRIPQAILDMIAGAHWGLLAGIAYFSMVGNWAKVLVVLLLFAGVDAETHVTGGS

AGHAAFGIANLFRSGPQQNIQLVNTDGSWHINRTALNCNDSLNTGWMAGLLYYNRFNSSGCTERLASCRP

LTSFAQGWGPISYTNGTGPDQRPYCWHYPPKPCGIVPAKSVCGPVYCFTPSPVVVGTTDRSGAPTYNWGD

NDTDVLVLNNTRPPLGNWFGCTWMNSTGFTKVCGAPPCAIGGVGNHTLLCPTDCFRKHPEATYSRCGSGP

WLTPRCLVNYPYRLWHYPCTINYTIFKVRMYVGGVEHRLEAACNWTRGERCNLEDRDRSELSPLLLSTTQ

WQVLPCSFTTLPALSTGLIHLHQNIVDVQYLYGVGSSIASWAIKWEYVVLLFLLLADARVCSCLWMMLLV

>gi|262387250|gb|ACY65363.1| envelope protein 2 [Hepatitis C virus subtype 1a]

MMMNWSLTTALVVAQLLRIPQAILDMIAGAHWAVLAGIAYFSMVGNWAKVLVVLLLFAGVDAETHVTGGS

AGHAAFGIANLFRSGPQQNIQLVNTNGSWHINRTALNCNDSLNTGWIAGLLYYNRLNSSGCTERLASCRP

LTSFAQGWGPISYTNGTGPDQRPYCWHYPPKPCGIVPAKSVCGPVYCFTPSPVVVGTTDRSGAPTYNWGD

NDTDVLVLNNTRPPLGNWFGCTWMNSTGFTKVCGAPPCAIGGVGNHTLLCPTDCFRKHPEATYSRCGSGP

WLTPRCLVNYPYRLWHYPCTINYTIFKVRMYVGGVEHRLEAACNWTRGERCNLEDRDRSELSPLLLSTTQ

WQVLPCSFTTLPALSTGLIHLHQNIVDVQYLYGVGSSIASWAIKWEYVVLLFLLLADARVCSCLWMMLLI

>gi|262387246|gb|ACY65361.1| envelope protein 2 [Hepatitis C virus subtype 1a]

MMMNWSPTTALVVAQLLRIPQAILDMIAGAHWGVLAGIAYFSMVGNWAKVLVVLLLFAGVDAETHVTGGS

AGHAAFGIANLFRSGPQQNIQLVNTNGSWHINRTALNCNDSLNTGWIAGLLYYDRFNSSGCTERLASCRP

LTSFAQGWGPISYTNGTGPDQRPYCWHYPPKPCGIVPAKSVCGPVYCFTPSPVVVGTTDRSGAPTYNWGD

NDTDVLVLNNTRPPLGNWFGCTWMNSTGFTKVCGAPPCAIGGVGNHTLLCPTDCFRKHPEATYSRCGSGP

WLTPRCLVNYPYRLWHYPCTINYTIFKVRMYVGGVEHRLEAACNWTRGERCDLEDRDRSELSPLLLSTTQ

WQVLPCSFTTLPALSTGLIHLHQNIVDVQYLYGVGSSIASWAIKWEYVVLPFLLLADARVCSCLWMMLLI

>gi|262387242|gb|ACY65359.1| envelope protein 2 [Hepatitis C virus subtype 1a]

MMMNWSPTTALVVAQLLRIPQAILDMIAGAHWGVLAGIAYFSMVGNWAKVLVVLLLFAGVDAETHVTGGS

AGHAAFGIANLFRSGPQQNIQPVNTNGSWHINRTALNCNDSLNTGWIAGLLYYNRSNSSGCTERLASCRP

LTSFAQGWGPISYTNGTGPDQRPYCWHYPPKPCGIVPAKSVCGPVYCFTPSPVVVGTTDRSGAPTYNWGD

NDTDVLVLNNTRPPLGNWFGCTWMNSTGFTKVCGAPPCAIGGVGNHTLLCPTDCFRKHPEATYSRCGSGP

WLTPRCLVNYPYRLWHYPCTINYTIFKVRMYVGGVEHRLEAARNWTRGERCNLEDRDRSELSPLLLSTTQ

WQVLPCSFTTLPALSTGLIHLHQNIVDVQYLYGVGSSIASWAIKWEYVVLLFLLLADARVCSCLWMMLLI

>gi|262387238|gb|ACY65357.1| envelope protein 2 [Hepatitis C virus subtype 1a]

MMMNWSPTTALEGALLLRIPQAILDMIAGAHWGVLAGIAYFSMVGNWAKVLVVLLLFAGVDAETHVTGGS

AGHAAFGIANLFRSGPQQNIQLVNTNGSWHINRTALNCNDSLNTGWIAGLLYYNRFNSSGCTERLASCRP

LTSFAQGWGPISYTNGTGPDQRPYCWHYPPKPCGIVPAKSVCGPVYCFTPSPVVVGTTDRSGAPTYNWGD

NDTDVLVLNNTRPPLGNWFGCTWMNSTGFTKVCGAPPCAIGGVGNHTLLCPTDCFRKHPEATYSRCGSGP

WLTPRCLVNYPYRLWHYPCTINYTIFKVRMYVGGVEHRLEAACNWTRGERCNLEDRDRSELSPLLLSTTQ

WQVLPCSFTTLPALSTGLIHLHQNIVDVQYLYGVGSSIASWAIKWEYVVLLFLLLADARVCSCLWMMLLV

>gi|262387234|gb|ACY65355.1| envelope protein 2 [Hepatitis C virus subtype 1a]

MMMNWSLTTALVVAQLLRIPQAILDMIAGAHWALLAGIAYFSMVGNWAKVLVVLLLFAGVDAETHVTGGS

AGHAAFGIANLFRSGPQQSIQLVNTNGSWHINRTALNCNDSLNTGWIAGLLYYNRFNSSGCTERLASCRP

LTSFAQGWGPISYTNGTGPDQRPYCWHYPPKPCGIVPAKSVCGPVYCFTPSPVVVGTTDRSGAPTYNWGD

NDTDVLVLNNTRPPLGNWFGCTWMNSTGFTKVCGAPPCAIGGVGNHTLLCPTDCFRKHPEATYSRCGSGP

WLTPRCLVNYPYRLWHYPCTINYTIFKVRMYVGGVEHRLEAACNWTRGERCNLEDRGRSELSPLLLSTTQ

WQVLPCSFTTLPALSTGLIHLHQNIVDVQYLYGVGSSIASWAIKWEYVVLLFLLLADARVCSCLWMMLLI

>gi|262387022|gb|ACY65249.1| envelope protein 2 [Hepatitis C virus subtype 1a]

MMMNWSPTAALVVAQLLRIPQAILDMIAGAHWAVLAGVAYFSMVGNWLKVLVVLLLFSSVDADTHVTGGT

VAHASHIITRLLTTGPKQNIQLINTNGSWHINRTALNCNASLNAGWIAGLFYYHKFNSSGCVERMASCKP

LADFDQGWGPISYANGTGPEHRPYCWHYPPKPCGIVPAQSVCGPVYCFTPSPVVVGTTNKLGAPTYNWGS

NDTDVFILNNTRPPSGNWFGCTWMNSTGFTKVCGAPPCNIGGVGNNTLHCPTDCFRKHPEATYSRCGSGP

WITPRCLVHYSYRLWHYPCTLNYTLFKVRMYVGGVEHRLDVACNWTRGERCDLDDRDRSELSPLLLSTTQ

WQVLPCSFTTLPALTTGLIHLHQNIVDVQYLYGVGSSIVSWAIKWEYVILLFLLLADARICSCLWMMLLI

>gi|262387018|gb|ACY65247.1| envelope protein 2 [Hepatitis C virus subtype 1a]

MMMNWSPTAALVVAQLLRIPQAILDMIAGAHWGVLAGIAYFSMVGNWPKGLVVLLLFPSVDADTHVTGGT

VAHASHIITRLLTTGPKQNIQLINTNGSWHINRTALNCNASLDAGWIAGLFYYHKFNSSGCVERMASCKP

LADLDQGWGPISYANGTGPEHRPYCWHYPPKPCGIVPAQSVCGPVYCFTPSPVVVGTTNKLGAPTYNWGS

NDTDVFILNNTRPPSGNWFGCTWMNSTGFTKVCGAPPCNIGGVGNNTLHCPTDCFRKHPEATYSRCGSGP

WITPRCLVHYSYRLGHYPCTLNYTLFKVRMYVGGVEHRLDVACNWTRGERCDLDDRDRSELSPLLLSTTQ

WQVLPCSFTTLPALTTGLIHLHQNIVDVQYLYGVGSSIVSWAIKWEYVILLFLLLADARICSCLWMMLLI

>gi|262387014|gb|ACY65245.1| envelope protein 2 [Hepatitis C virus subtype 1a]

MMMNWFPTAALVVAQLLRIPQAILDMIAGAHWGVLAGVAYFSMVGNWAKVLVVLLLFSSVDADTHVTGGT

VAHASHIITRLLTTGPKQNIQLINTNGSWHINRTALNCNASLDAGWTAGLFYYHKFNSSGCVERMASCKP

LADFDQGWGPISYANGTGPEHRPYCWHYPPKPCGIVPAQSVCGPVYCFTPSPVVVGTTNKLGAPTYSWGS

NDTDVFILNNTRPPSGNWFGCTWMNSTGFTKVCGAPPCNIGGVGNNTLHCPTDCFRKHPEATYSRCGSGP

WITPRCLVHYSYRLWHYPCTLNYTLFKVRMYVGGVEHRLDVACNWTRGERCDLDDRDRSELSPLLLSTTQ

WQVLPCSFTTLPALTTGLIHLHQNIVDVQYLYGVGSSIVSWAIKWEYVILLFLLLADARICSCLWMMLLV

>gi|262387010|gb|ACY65243.1| envelope protein 2 [Hepatitis C virus subtype 1a]

MMMNWSPTAALVVAQLLRIPQAILDMIAGAHWGVLAGVAYFSMVGNWAKVLVVLLLFSSVDADTHVTGGT

VAHASHIITRLLTTGPKQNIQLINTNGSWHINRTALNCNASLDAGWIAGLFYYHKFNSSGCVERMASCKP

LADFDQGWGPISYANGTGPEHRPYCWHYPPKPCGIVPAQSVCGPVYCFTPSPVVVGTTNKLGAPTYNWGS

NDTDVFILNNTRPPSGNWFGCTWMNSTGFTKVCGAPPCNIGGVGNNTLHCPTDCFRKHPEATYSRCGSGP

WITPRCLVHYSYRLWHYPCTLNYTLFKVRMYVGGVEHRLDVACNWTRGERCDLDDRDRSELSPLLLSTTQ

WQVLPCSFTTLPALTTGLIHLHQNIVDVQYLYGVGSSIVSWAIKWEYVILLFLLLADARICSCLWMMLLI

>gi|262387006|gb|ACY65241.1| envelope protein 2 [Hepatitis C virus subtype 1a]

MMMNWSPTAALVVGKLLKIPQAILDMIAGAHWGVLAGVAYFSMVGNCAKVLVVLLLFSSVDADTHVTGGT

VAHASHIITRLLTTGPKQNIQLINTNGSWHINRTALNCNASLDAGWIAGLFYYHKFNSSGCVERMASCKP

LADFDQGWGPISYANGTGPEHRPYCWHYPPKPCGIVPAQSVCGPVYCFTPSPVVVGTTNKLGALTYNWGS

NDTDVFILNNTRPPSGNWFGCTWMNSTGFTKVCGAPPCNIGGVGNNTLHCPTDCFRKHPEATYSRCGSGP

WITPRCLVHYSYRLWHYPCTLNYTLFKVRMYVGGVEHRLDVACNWTRGERCDLDDRDRSELSPLLLSTTQ

WQVLPCSFTTLPALTTGLIHLHQNIVDVQYLYGVGSSIVSWAIKWEYVILLFLLLADARICSCLWMMLLV

>gi|262387384|gb|ACY65430.1| envelope protein 2 [Hepatitis C virus subtype 1a]

MMMNWSPTTALVVAQLLRIPQAILDMIAGAHWGVLAGIAYFSMVGNWAKVVVVLLLFAGVDAETHTTGAV

AGRVTSSFTSVFTQGAKQNIQLINSNGSWHINRTALNCNDSLSTGWLAGLFYYSKFNSSGCPERMASCRP

LADFAQGWGPIRYANGSGPDHRPYCWHYPPRPCGIVPARSVCGPVYCFTPSPVAVGTTDGSGVPTYSWGE

NETDVFILNNTRPPQGNWFGCTWMNSTGYTKVCGAPPCDIGGVGNNTLRCPTDCFRKHPEATYSRCGSGP

WLTPRCLVDYPYRLWHYPCTVNYSIFKVRMYVGGVEHRLEAACNWTRGERCDLEDRDRSELSPLLLSTTQ

WQVLPCSFTTLPALSTGLIHLHQNIVDVQYLYGVGSSIASWAIKWEYVVLLFLLLADARVCSCLWMMLLV

>gi|262387380|gb|ACY65428.1| envelope protein 2 [Hepatitis C virus subtype 1a]

MMMNWSPTTALVVAQLLRIPQAILDMIAGAHWGVLAGIAYFSMVGNWAKVVVVLLLFAGVDAETHTTGAV

AGRVTSSFTSFLMQGPKQNVQLINSNGSWHINRTALNCNDSLSTGWVAGLFYYSKFNSSGCPERMASCRP

LADFAQGWGPIRYANGSGPDHRPYCWHYPPRPCGIVPARSVCGPVYCFTPSPVAVGTTDGSGVPTYSWGE

NETDVFILNNTRPPQGNWFGCTWMNSTGYTKVCGAPPCDIGGVGNNTLRCPTDCFRKHPEATYSRCGSGP

WLTPRCLVDYPYRLWHYPCTVNYSIFKVRMYVGGVEHRLEAACNWTRGERCDLEDRDRSEPSPLLLSTTQ

WQVLPCSFTTLPALSTGLIHLHQNIVDVQYLYGVGSSIASWAIKWEYVVLLFLLLADARVCSCLWMMLLV

>gi|262387376|gb|ACY65426.1| envelope protein 2 [Hepatitis C virus subtype 1a]

MMMNWSPTTALVVAQLLRIPQAILDMIAGAHWGVLAGIAYFSMVGNWAKVVVVLLLFAGVDAETHTTGAV

AGRVTSSFTSFFMQGPKQNIQLINSNGSWHINRTALNCNDSLSTGWVAGLLYYSKFNSSGCPERMASCRP

LADFAQGWGPIRYANGSGPDHRPYCWHYPPRPCGIVPARSVCGPVYCFTPSPVAVGTTDRSGVPTYNWGG

NETDVFILNNTRPPQGNWFGCTWMNSTGYTKVCGAPPCDIGGVGNNTLRCPTDCFRKHPEATYSRCGSGP

WLTPRCLVDYPYRLWHYPCTVNYSIFKVRMYVGGVEHRLEAACNWTRGERCDLEDRDRSELSPLLLSTTQ

WQVLPCSFTTLPALSTGLIHLHQNIVDVQYLYGVGSSIASWAIKWEYVVLLFLLLADARVCSCLWMMLLV

>gi|262387372|gb|ACY65424.1| envelope protein 2 [Hepatitis C virus subtype 1a]

MMMNWSLTTALVVAQLLRIPQAILDMIAGAHWGVLAGIAYFSMVGNWAKVVVVLLLFAGVDAETHTTGAV

AGRVTSSFTSFFMQGPKQNIQLINSNGSWHINRTALNCNDSLSTGWVAGLLYYSKFNSSGCPERMASCRP

LADFAQGWGPIRYANGSGPDHRPYCWHYPPKPCGIVPARSVCGPVYCFTPSPVAVGTTDRSGVPTYNWGG

NETDVFILNNTRPPQGNWFGCTWMNSTGYTKVCGAPPCDIGGVGNNTLRCPADCFRKHPEATYSRCGPGP

WLTPRCLVDYPYRLWHYPCTVNYSIFKVRMYVGGVEHRLEAACNWTRGERCDLEDRDRSELSPLLLSTTQ

WQVLPCSFTTLPALSTGLIHLHQNIVDVQYLYGVGSSIASWAIKWEYVVLLFLLLADARVCSCLWMMLLI

>gi|262387368|gb|ACY65422.1| envelope protein 2 [Hepatitis C virus subtype 1a]

MMMNWSPTTALVVAQLLRIPQVILDMIAGAHWGVLAGIAYFSMVGNWAKVVVVLLLFAGVDAETHTTGAV

AGRVTSSFTSFFMQGPKQNIQLINSNGSWHINRTALNCNDSLSTGWVAGLLYYSKFNSSGCPERMASCRP

LADFAQGWGPIRYANGSGPDHRPYCWHYPPRPCGIVPARSVCGPVYCFTPSPVAVGTTDGSGVPTYNWGE

NETDVFILNNTRPPQGNWFGCTWMNSTGYTKVCGAPPCDIGGVGNNTLRCPTDCFRKHPEATYSRCGSGP

WLTPRCLVDYPYRLWHYPCTVNYSIFKVRMYVGGVEHRLEAACNWTRGERCDLEDRDRSELSPLLLSTTQ

WQVLPCSFTALPALSTGLIHLHQNIVDVQYLYGVGSSIASWAIKWEYVVLLFLLLADARVCSCLWMMLLI

>gi|262387364|gb|ACY65420.1| envelope protein 2 [Hepatitis C virus subtype 1a]

MMMNWSPTTALVVAQLLRIPQAILDMIAGAHWGVLAGIAYFSMVGNWAKVVVVLLLFAGVDAETHTTGAV

AGRVTSSFTSFFMQGPKQNIQLINSNGSWHINRTALNCNDSLSTGWVAGLLYYSKFNSSGCPERMASCRP

LADFAQGWGPIRYANGSGPDHRPYCWHYPPRPCGIVPARSVCGPVYCFTPSPVAVGTTDGSGVPTYSWGE

NETDVFILNNTRPPQGNWFGCTWMNSTGYTKVCGAPPCDIGGVGNNTLRCPTDCFRKHPEATYSRCGSGP

WLTPRCLVDYPYRLWHYPCTVNYSIFKVRMYVGGVEHRLEAACNWTRGERCDLEDRDRSEPSPLLLSTTQ

WQVLPCSFTTLPALSTGLIHLHQDIVDVQYLYGVGSSIASWAIKWGYVVLLFLLLTDARVCSCLWMMLLI

>gi|262387360|gb|ACY65418.1| envelope protein 2 [Hepatitis C virus subtype 1a]

MMMNWSPTTALVVAQLLRIPQAILDMIAGAHWGVLAGIAYFSMVGNWAKVVVVLLLFAGVDAETHTTGAV

AGRVTSSFTSFFMQGPKQNIQLINSNGSWHINRTVLNCNDSLSTGWVAGLFYYSKFNSSGCPERMASCRP

LADFAQGWGPIRYANGSGPDHRPYCWHYPPKPCGIVPARSVCGPVYCFTPSPVAVGTTDGSGVPTYNWGD

NETDVFILNNTRPPQGNWFGCTWMNSTGYTKVCGAPPCDIGGVGNNTLRCPTDCFRKHPEATYSRCGSGP

WLTPRCLVDYPYRLWHYPCTVNYSIFKVRMYVGGVEHRLEAACNWTRGERCDLEDRDRSELSPLLLSTTQ

WQVLPCSLTALPAPSTGLIHLHQNIVDVQYLYGVGSSIASWAIKWEYVVLLFLLLADARVCSCLWMMLLI

>gi|262387356|gb|ACY65416.1| envelope protein 2 [Hepatitis C virus subtype 1a]

MMMNWSPTTALVVAQLLRIPQAILDMIAGAHWGVLAGIACFSMVGNWAKVVVVLLLFAGVDAETHTTGAV

AGRVTSSFTSFFMQGPKQNIQLINSNGSWHINRAALNCNDSLSTGWVAGLLYYSKFNSSGCPERMASCRP

LADFAQGWGPIRYANGSGPDHRPYCWHYPPRPCGIVPARSVCGPVYCFTPSPVAVGTTDGSGVPTYSWGE

NETDVFILNNTRPPQGNWFGCTWMNSTGYTKVCGAPPCDIGGVGNNTLCCPTDCFRKHPEATYSRCGSGP

WLTPRCLVDYPYRLWHYPCTVNYSIFKVRMYVGGVEHRLEAACNWTRGERCDLEDRDRSELSPLLLSTTQ

WQVLPCSFTTLPALSTGLIHLHQNIVDVQYLYGVGSSIASWAIKWEYVVLLFLLLADARVCSCLWMMLLI

>gi|262387352|gb|ACY65414.1| envelope protein 2 [Hepatitis C virus subtype 1a]

MMMNWSPTTALVVAQLLRIPQAILDMIAGAHWGVLAGVAYFSMVGNWAKVVVVLLLLAGVDAETHTTGAV

AGRVTSSFTSFFMQGPKQNIQLINSNGSWHINRTALNCNDSLSTGWVAGLLYYSKFNSSGCPERMASCRP

LADFAQGWGPIRYANGSGPDHRPYCWHYPPKPCGIVPARSVCGPVYCFTPSPVAVGTTDRSGVPTYNWGG

NETDVFILNNTRPPQGNWFGCTWMNSTGYTKVCGAPPCDIGGVGNNTLRCPTDCFRKHPEAIYSRCGSGP

WLTPRCLVDYPYRLWHYPCTVNYSIFKVRMYVGGVEHRLEAACNWTRGERCDLEDRDRSELSPLLLSTTQ

WQVLPCSFTTLPALSTGLIHLHQNIVDVQYLYGVGSSIASWAIKWEYVVLLFLLLADARVCSCLWMMLLV

>gi|262387348|gb|ACY65412.1| envelope protein 2 [Hepatitis C virus subtype 1a]

MMMNWSPTTALVVAQLLRIPQAILDMTAGAHWGVLAGIAYFSMVGNWAKVVVVLLLFAGVDAETHTTGAV

AGRVTSSFTSFFMQGPKQNIQLINSNGSWHINRTALNCNDSLSTGWVAGLLYYSKFNSSGCPERMASCRP

LADFAQGWGPIRYANGSGPDHRPYCWHYPPRPCGIVPARSVCGPVYCFTPSPVAVGTTDGSGVPTYNWGE

NETDVFILNNTRPPQGNWFGCTWMNSTGYTKVCGAPPCDIGGVGNNTLRCPTDCFRKHPEATYSRCGSGP

WLTPRCLVDYPYRLWHYPCTVNYSIFKVRMYVGGVEHRLEAACNWTRGERCDLEDRDRSELSPLLLSTTQ

WQVLPCSFTALPALSTGLIHLHQNIVDVQYLYGVGSSIASWAIKWEYVVLLFLLLADARVCSCLWMMLLI

>gi|262387268|gb|ACY65372.1| envelope protein 2 [Hepatitis C virus subtype 1a]

MMMNWSPTTALVVAQLLRIPQAILDMIAGAHWGVLAGIAYFSMVGNWAKVLVVLLLFAGVDAETHVTGGS

AGHAAFGIANLFRSGPQQNIQLVNTNGSWHINRTALNCNDSLNTGWIAGLLYYNRFNSSGCTERLASCRP

LTSFAQGWGPISYTNGTGPDQRPYCWHYPPKPCGIVPAKSVCGPVYCFTPSPVVVGTTDRSGAPTYNWGD

NDTDVLVLNNTRPPLGNWFGCTWMNSTGFTKVCGAPPCAIGGVGNHTLLCPTDCFRKHPEATYSRCGSGP

WLTPRCLVNYPYRLWHYPCTINYTIFKVRMYVGGVEHRLEAACNWTRGERCNLEDRDRSELSPLLLSTTQ

WQVLPCSFTTLPALSTGLIHLHQNIVDVQYLYGVGSSIASWAIKWEYVVLLFLLLADARVCSCLWMMLLV

>gi|348609325|gb|AEP71358.1| E2 [synthetic construct]

MASTYTSGGSAARDVHGLVGLFSPGAHQRLQLVNTNGSWHINRTSLNCNDSINTGFTAGLFYYHKFNSIG

CPQRLSSCKPITFFKQGWGPLTDANITGSSNDRPYCWHYPPRPCEVVRASSVCGPVFCFTPSPVVVGTTD

VVGAPTYSWGENETDVFLLTSLPPPAGRWFGCMWMNCTGFVKTCGAPPCNIYGGEKGSNPHNESDLFCPT

DCFRKHREATYSRCGAGPWLTPRCLVDYPYRLWHYPCTVNFTLFKVRMFVAGFEHRFQAACNWTRGERCD

IEDRDRSELHPLLHSTTELAILPCSFTPMPALSTGLIHLHQNIVDVQYLYGVGAGMVGWGVKWEFVILFS

LLLAFAE

**Genotype 3a Sequences:**

>gi|359430951|gb|AEV46286.1| polyprotein [Hepatitis C virus genotype 3]

MSTLPKPQRKTKRNTIRRPQDVKFPGGGQIVGGVYVLPRRGPRLGVRATRKTSERSQPRGRRQPIPKARR

SEGRSWAQPGYPWPLYGNEGCGWAGWLLSPRGSRPTWGPNDPRRRSRNLGKVIDTLTCGFADLMGYIPVV

GAPVGGVARAFAHGVRALEDGINFATGNLPGCSFSIFLLALLSCLVHPAAGFEWRNTSGLYVLTNDCPNS

SIVYEADDVILHTPGCVPCVHADNTSTCWTPVTPTVAVRYPGATTASVRSHVDLLVGAATMCSALYVGDM

CGAVFLVGQAFTFKPRRHQTVQTCNCSLYPGHITGHRMAWDMMMNWSPAVGLVVAHVLRLPQTLLDIIAG

AHWGILAGLAYYSMQGNWAKVAIIMVMFSGVDAGTHTTGGAVAYKTNLFASLFNAGSKQNVQLINTNGSW

HINRTALNCNDSINTGFLAGLFYYHKFNCTGCPQKLSSCEPITSFNQGWGPLKDANITGHNSTEKPYCWH

YAPNPCDTVVAKNVCGPVYCFTPSPVVVGTTDKRGVPTYTWGENETDVFLLRSLRPPSGQWFGCVWMDSM

GFLKTCGAPPCDIYGGMKAWKDRDRSDLFCPTDCFRKHPGATYSRCGAGPWLTPRCMVDYPYRLWHYPCT

VNYTLFKVRMYVGGIEHRFSAACNWTRGERSDIEDRDRSEQHPLLHSTTELAMLPSLPALLTGLIHLHQN

IVDVQYLYGVGAGTVGWALKWEFVILVFLLLADARVCVALWLMLMISQAEAALENLVTLNAIAAAGTHGA

GWYLVAFCAAWYVRGKLVPLVTYSLTGLWSLALLVLLLPQRAYAWSGEDSATLGAGVLVLFGFFTLSPWY

KHWIGRLMWWNQYAICRCEAALHVWVPPQLARGSRDGVILLTSLFYPSLIFDITKLLIAILGPLYLIQAA

ITATPYFVRAHVLVRLCMLVRSVMGGKYFQMVILSIGRWFNTYLYDHLAPMQHWAASGLRDLAVATEPVI

FSPMEIKVITWGADTAACGDILCGLPVSARLGHEVLLGPADNYREMGWRLLAPITAYAQQTRGLLGTIVT

SLTGRDKNVVTGEVQVLSTTTQTFLGTTVGGVMWTVYHGAGSRTLAGVKHPALQMYTNVDQDLVGWPAPP

GAKSLEPCACGSADLYLVTRDADVIPARRRGDSTASLLSPRPLACLKGSSGGPVMCPSGHVAGIFRAAVC

TRGVAKALQFIPVETLSTQARSPSFSDNSTPPAVPQSYQVGYLHAPTGSGKSTKVPAAYVAQGYNVLALN

PSVAATLGFGSFMSRAYGIDPNIRTGTRTVTTGAKLTYSTYGKFLADGGCSGGAYDVIICDECHAQDATS

ILGIGTVLDQAETAGVRLTVLATATPPGSITVPHSNIEEVALGSEGEIPFYGKAIPMALLKGGRHLVFCH

SKKKCDEIASKLRGMGLNAVAYYRGLDVSVIPTTGDVVVCATDALMTGYTGGFDSVIDCNVAVEQYVDFS

LLDPTFSIETRTAPQDAVSRSQRRGRTGRGRLGTYRYVAPGERPSGMFDSVVLCECYDAGCSWYDLQPAE

TTVRLRAYLSTPGLPVCQDHLDFWESVFTGLTHIDAHFLSQTKQQGLNFSYLTAYQATVCARAQAPPPSW

DETWKCLVRLKPTLHGPTPLLYRLGPIQNEICLTHPITKYIMACMSADLEVTTSTWVLLGGVLAALAAYC

LSVGCVVIVGHIELGGMPALVPDKEVLYQQYDEMEECSQAVPYIEQAQVIAHQFREKVLGLLQRATQQQA

VIEPIVATNWQKLEAFWHKHMWNFVSGIQYLAGLSTLPGNPAVASLMAFTASVTSPLTTNQTMFFNILGG

WVATHLAGPQSSSAFVVSGLAGAAIGGIGLGRVLLDILAGYGAGVSGALAAFKIMGGDLPTTEDMVNLLP

AILSPGALVVGVICAAILRRHVGPGEGAVQWMNRLIAFASRGNHVSPTHYVPESDVAARITALLSSLTVT

RLLRRLHQWINEDYPSPCSGDWLRTIWDWVCMVLSDFKTWLSSKIMPALPGLPFISCQEGYKGVWRGDGV

MSTRCPCGASITGHVKNGSMRLAGPRICANMWHGTFPINEYTTGPSTPCPSPNYTRALWRVAANSYVEVR

RVGDFHYITGATEDELKCPCQVPAAEFFTEVDGVRIHRYAPPCKPLLRDEITFTVWGCIPMRSQLPCEPE

PDVSVLTSMLRDPSHITAETAARRLARGSPPSEASSSASQLSAPSLKATCQTHRPHPDAELVDANLLWRQ

EMGSNITRVESETKVVVLDSFEPLRAETDDVEPSVAAECFKKPPKYPPALPIWARPDYNPPLLDRWKAPD

YVPPTVHGCALPPRGAPPVPPPRRKRTIQLDGSNVSAALAALAEKSFPSSKPQEENSSSSGVDTQSSTTS

KVPPSPGGESDSESCSSMPPLEGEPGDPDLSCDSWSTVSDSEEQSVVCCSMSYSWTDALITPCSAEGEKL

PISPLSNSLERHHNLIYSTSSRIASQRQKKVTFDRLQVLDDHYKTALKEIKERASRVKARMLTIEEACAL

VPPHSARSKFGYSAKDARSLSSKAINQIRSVWEDLLEDTTTPIPTTIMAKSEVFCVDPTKGGRFFARLIV

YPDLGVRICEKRALYGVIQKWGVGTMGPAYGFQYSPQQRVERLLKMWTSKKAQLGFSYGTRCFGSTATGQ

DIRVEEEIYQCWSLGPEARKVISSLTERLYCGGPMFNSKGAQCGYRRCRASGVLPTSFGNTITCYIKATA

AAKAANLRKPGFLFGEDGSWSYYLRTECDGVDEDRATLRAFTEAMTRYSAPPGDAPQATYDLELITSCSS

NVSVARDDKGRRYYYLTRDATTPLARAAWETARHTPVTSWLGNIIMYAPTIWVRMVMMTHFFSILQSQEI

LDRPLDFEMYGATYSVTPLDLPAIIGRLHGLRAFTLHIYSPAELNTVAGTLRKLGCPPLRAWRHRARAVR

AMLIAQGGKARICGLYHFNWAVRTKTTLTPLPAAGQLDLSIWFTVGVGGNDILAACHAPEPAICCFAYSL

LTVGVGIFLLPAR

>gi|166159805|gb|ABY83294.1| polyprotein [Hepatitis C virus subtype 3a]

HTYTTGGTAARHTQAFAGLFDIGPQQKLQLINTYGSGHINRTAPNCNESIKHRFLAGLFYYHKFNLTGCP

QRLSRCKAITFFRQGWGPLTDANITGPSDDKPYCWQYAPRPCDTVKQATVCGPGYCFTPSPVGVGTTDPK

GGPTYNWGGDETDVFLLKSLRPPSGRWFGCTWMNSTGFVKTCGGPPCDIYGGGGRSTNGSDLFCPTDCFR

KHPEATYSRCGSGPWLTPRCMVDYPYRLWHYPCTVNFTLFKVRMFVGGFEHRFTAACNWTRGERCDIEDR

DRSEQHPLLHSTTELAILPCSFTPMPALSTGLIHLHQNIVDVQYLYGVGSGMVGWALKWEFVILVFLLLA

DA

>gi|166159807|gb|ABY83295.1| polyprotein [Hepatitis C virus subtype 3a]

LEWRNTSGLYVLTNDCPNSSIVYEADDVILHTPGCIPCVQDGNISRCWTPVTPTVAVRYVGATTASVRSH

VDLLVGAATMCSALYEGDMWGAVFLVGQAFTFRPRRHQTVQTCNCSLYPDHLSGHRMAWDMMMNWSPAVG

MVVAHVLRLPQTLFDILAGAHWGILAGLAYYSMQGNWDKVGIILVMFSGVDADTYITGGTAARHTQAFAG

LFDIGPQQKLQLVNTYGSLHISRTAPTCNESINTGFLAGLFYYHKFNSTGCPRRLSSCKPITFFRQGWGP

LTDANITGPSDDRPYCWHYAPRPCDTVKAATVCGPVYCFTPSPVVVGTTDPMGAPTYTWGENETDVFLLK

SLRPPSGRWFGCTWMNYTGFVKTCGGPPCDIYGGGGRSTNGSDLFCPTDCFRKHPEATYSRCGSGPWLTP

RCMVDYPYRLWHYPCTVNFTLFKVRMFVGGFEHRFTAACNWTRGERCDIEDRDRSEQHPLLHSTTELAIL

PCSFTPMPALSTGLIHLHQNIVDVQYLYGDGSGMVGWALKWEFVILVFLLLADA

>gi|310705003|gb|ADP08269.1| envelope protein 2 [Hepatitis C virus subtype 3a]

QTHLTGSSIAYSTRGLTSLFTSGAKQKLQLVNTNGSWHINSTALNCNDSLNTGFLAGLLYHHKFNSTGRP

QRLSSCKPLAFFRQGWGSLTDANITGPSNDRPYCWHYAPRPCGIVLALNVCGPVYCFTPSPVVVGTTDAK

GAPTYTWGENETDVFLLKSLRPPSGRWFGCVWMNSTGFLKTCGAPPCNIYGVGGDSNNESDLFCPTDCFR

KHPEATYSRCGAGPWLTPRCMVDYPYRLWHFPCTVNFTLFKVRMFVGGFEHRFSAACNWTRGERCDIEDR

DRSEQQPLLHSTTELAILPCSFTPMPALSTGLIHLHQNIVDVQYLYGVGSGMVGWALKWEFVILVFLLLA

DA

>gi|310705005|gb|ADP08270.1| envelope protein 2 [Hepatitis C virus subtype 3a]

HTYITGSNIAHNTRGLASLFTSGAKQKLQLVNTNGSWHINSTALNCNDSLNTGFLAGLLYHHKFNSTGCP

QRLSSCKPLAFFRQGWGSLTDANITGPSNDRPYCWHYAPRPCGIVPALNVCGPVYCFTPSPVVVGTTDAK

GAPTYTWGENETDVFLLKSCGPPSGRWFGCVWMNSTGFLKTCGAPPCNIYGVGGIPNNESDLFCPTDCFR

KHPEATYSRCGAGPWLTPRCMVDYPYRLWHFPCTVNFTLFKVRMFVGGFAHRFSAACNWTRGERCDIEDR

DRSEQQPLLHSTTELAILPCSFTPMPALSTGLIHLHQNIVDVQYLYGVGSGMVGWALKWEFVILVFLLLA

DA

>gi|310705001|gb|ADP08268.1| envelope protein 2 [Hepatitis C virus subtype 3a]

QTHPTGSSIAYSTRGLTSLFTSGAKQWLQLVNTNGSWHINSTALNCNDSLNTGFLAGLLYHHKFNSTGCP

QRLSSCKPLAFFRQGWGSLTDANITGPSNDRPYCWHYAPRPCGIVPALNVCGPVYCFTPSPVVVGTTDAR

GAPTYTWGENETDVFLLKSCGPPSGRWFGCVWMNSTGFLKTCGAPPCNIYGVGGIPNNESDLFCPTDCFR

KHPEATCSRCGAGPWLTPRCMVDYPYRLWHFPCTVNFALFKVRMFVGGFEHRFSAACNWTRGERCDIEDR

DRSEQQPLLHSTTELAILPCSFTPMPALSTGLIPLHQNIVDVQYLYGVGSGMVGWALKWEFVILVFLLLA

DA

>gi|410069937|gb|AFV59190.1| envelope glycoprotein E2, partial [Hepatitis C virus]

QTHVTGGSAAREARGSTSLFSLGASQKLQLVNTNGSWHINRTALNCNESINTGFIAGLFYYHKFNSTGCP

QRLSSCKPITSFEQGWGPITDANISGSSNDRPYCWHYAPRPCGVVNASSVCGPVYCFTPSPVVVGTTDAK

GVPTYTWGENETDVFLLESLRPPGGRWLGCAWMNSTGFLKTCGAPPCDIYGGSKKPDNNQALFCPTDCFR

KHPDATYSRCGAGPWITPRCMVDYPYRLWHYPCTVNFTLFKARMFVGGFEHRFSAACNWTRGERCNIEDR

DRSEQQPLLHSTTEFAILPCSFTPMPALSTGLIHLHQDIVDVQYLYGVGSGMVGWALKWEFVVLIFLLQA

DARVCVALWLMLMISQAEA

>gi|410069933|gb|AFV59188.1| envelope glycoprotein E2, partial [Hepatitis C virus]

QTHVTGGSAAREARGFTSLFSLGASQKLQLVNTNGSWHINRTALNCNESINTGFIAGLFYYHKFNSTRCP

QRLSSCKPITSFEQGWGPITDANISGSSNDRPYCWHYAPRPCGVVNASSVCGPVYCFTPSPVVVGTTDAK

GVPTYTWGENETDVFLLESLRPPGGRWLGCAWMNSTGFLKTCGAPPCDIYGGSKKPDNNQALFCPTDCFR

KHPDATYSRCGAGPWITPRCMVDYPYRLWHYPCTVNFTLFKARMFVGGFEHRFSAACNWTRGERCNIEDR

DRSEQQPLLHSTTELAILPCSFTPMPALSTGLIHHHQDIVDVQYLYGVGSGMVGWALKWEFVVLIFLLLA

DARVCVALWLMLMISQAEA

>gi|410069929|gb|AFV59186.1| envelope glycoprotein E2, partial [Hepatitis C virus]

QTHVTGGSAAREARGFTSLFSLGASQKLQLVNTNGSWHINRTALNCNESINTGFIAGLFYYHKFNSTGCP

QRLSSCKPITSFEQGWGPITDANISGSSNDRPYCWHYAPRPCGVVNASSVCGPVYCFTPSPVVVGTTDAK

GVPTYTWGENETDVFLLESLRPPGGRWLGCAWMNSTGFLKTCGAPPCDIYGGSKKPDNNQALFCPTDCFR

KHPDATYSRCGAGPWITPRCMVDYPYRLWHYPCTVNFTLFKVRMFVGGFEHRFSAACNWTRGERCNIEDR

DRSEQQPLLHSTTEFAILPCSFTPMPALSTGLIHLHQDIVDVQYLYGVGSGMVGWALKWEFVVLIFLLLA

DARVCVALWLMLMISQAEA

>gi|410069925|gb|AFV59184.1| envelope glycoprotein E2, partial [Hepatitis C virus]

QTHVTGGSAAREARGFTSLFSLGASQKLQLVNTNGSWHINRTALNCNESINTGFIAGLFYYHKFNSTGCP

QRLSSCKPITSFEQGWGPITDANISGSSNDRPYCWHYAPRPCGVVNASSVCGPVYCFTPSPVVVGTTDAK

GVPTYTWGENETDVFLLESLRPPGGRWLGCAWMNSTGFLKTCGAPPCDIYGGSKKPDNNQALFCPTDCFR

KHPDATYSRCGAGPWITPRCMVDYPYRLWHYPCTVNFTLFKVRMFVGGFEHRFSAACNWTRGERCNIEDR

DRSEQQPLLHSTTEFAILPCSFTPMPALSTGLIHLHQDIVDVQYLYGVGSGMVGWALKWEFVVLIFLLLA

DARVCVALWLMLMISQAES

>gi|410069921|gb|AFV59182.1| envelope glycoprotein E2, partial [Hepatitis C virus]

QTHVTGGSAAREARGFTSLFSLGASQKLQLVNTNGSWHINRTALNCNESINTGFIAGLFYYHKFNSTGCP

QRLSSCKPITSFEQGWGPITDANISGSSNDRPYCWHYAPRPCGVVNASSVCGPVYCFTPSPVVVGTTDAK

GVPTYTWGENETDVFLLESLRPPGGRWLGCAWMNSTGFLKTCGAPPCDIHGGSKKPDNNQALFCPTDCFR

KHPDATYSRCGAGPWITPRCMVDYPYRLWHYACTVNFTLFKVRMFVGGFEHRFSAACNWTRGERCNIEDR

DRSEQQPLLHSTTEFATLPCSFTPMPALSTGLIHLHQDIVDVQYLYGVGSGMVGWALKWEFVVLIFLLLA

DARVCVALWLMLMISQAEA

>gi|410069917|gb|AFV59180.1| envelope glycoprotein E2, partial [Hepatitis C virus]

QTHVTGGSAAREARGFTSLFSLGASQKLQLVNTNGSWHINRTALNCNESINTGFIAGLFYYHKFNSTGCP

QRLSSCKPITSFEQGWGPITDANISGSSNDRPYCWHYAPRPCGVVNASSVCGPVYRFTPSPVVVGTTDAK

GVPTYTWGENETDVFLLESLRPPGGRWLGCAWMNSTGFLKTCGAPPCDIYGGSKKPDNNQALFCPTDCFR

KHPDATYGRCGAGPWITPRCMVDYPYRLWHYPCTVNFTLFKVRMFVGGFEHRFSAACNWTRGERCNIEDR

DRSEQQPLLHSTTEFAILPCSFTPMPALSTGLIHLHQDIVDVQYLYGVGSGMVGWALKWEFVVLIFLLLA

DARVCVALWLMLMISQAEA

>gi|410069913|gb|AFV59178.1| envelope glycoprotein E2, partial [Hepatitis C virus]

QTHVTGGSAAREARGFTSLFSLGASQKLQLVNTNGSWHINRTALNCNESINTGFIAGLFYYHKFNSTGCP

QRLSSCKPITSFEQGWGPITDANISGSSNDRPYCWHYAPRPCGVVNASSVCGPVYCFTPSPVVVGTTDAK

GVPTYTWGENETDVFLLESLRPPGGRWLGCAWMNSTGFLKTCGAPPCDIYGGSKKPDNNQALFCPTDCFR

KHPDATYSRCGAGPWITPRCMVDYPYRLWHYPCTVNFTLFKVRMFVGGFEHRFSAACNWARGERCNIEDR

DRGEQQPLLHSTTEFAILPCSFTPMPALSTGLIHLHQDIVDVQYLYGVGSGMVGWALKWEFVVLIFLLLA

DARACVALWLVLMISQAEA

>gi|410069909|gb|AFV59176.1| envelope glycoprotein E2, partial [Hepatitis C virus]

QTHVTGGSAAREARGFTSLFSLGASQKLQLVNTNGSWHINRTALNCNESINTGFIAGLFYYHKFNSTRCP

QRLSSCKPITSFEQGWGPITDANISGSSNDRPYCWHYAPRPCGVVNASSVCGPVYCFTPSPVVVGTTDAK

GVPTYTWGENETDVFLLESPRPPGGRWLGCAWMNSTGFLKTCGAPPCDIYGGSKKPDNNQALFCPTDCFR

KHPDATYSRCGAGPWITPRCMVDYPYRLWHYPCTVNFTLFKVRMFVGGFEHRFSAACNWTRGERCNIEDR

DRSEQQPLLHSTTEFAILPCSFTPMPALSTGLIHLHQDIVDVQYLYGVGSGMVGWALKWEFVVLIFLLLA

DARVCVALWLMLMISQAEA

>gi|410069939|gb|AFV59191.1| envelope glycoprotein E2, partial [Hepatitis C virus]

QTHVTGGSAAREARGSTSLFSLGASQKLQLVNTNGSWHINRTALNCNESINTGFIAGLFYYHKFNSTGCP

QRLSSCKPITSFEQGWGPITDANISGSSNDRPYCWHYAPRPCGVVNASSVCGPVYCFTPSPVVVGTTDAK

GVPTYTWGENETDVFLLESLRPPGGRWLGCAWMNSTGFLKTCGAPPCDIYGGSKKPDNNQALFCPTDCFR

KHPDATYSRCGAGPWITPRCMVDYPYRLWHYPCTVNFTLFKVRMFVGGFEHRFSAACNWTRGERCNIEDR

DRSEQQPLLHSTTEFAILPCSFTPMPALSTGLIHLHQDIVDVQYLYGVGSGMVGWALKWEFVVLIFLLLA

DARLCVALWLMLMISQAEA

>gi|410069935|gb|AFV59189.1| envelope glycoprotein E2, partial [Hepatitis C virus]

QTHVTGGSAAREARGFTSLFSLGASQKLQLVNTNGSWHINRTALNCNESINTGFIAGLFYYHKFNSTRCP

QRLSSCKPITSFEQGWGPITDANISGSSNDRPYCWHYAPRPCGVVNASSVCGPVYCFTPSPVVVGTTDAK

GVPTYTWGENETDVFLLESLRPPGGRWLGCAWMNSTGFLKTCGAPPCDIYGGSKKPDNNQALFCPTDCFR

KHPDATYSRCGAGPWITPRCMVDYPYRLWHYPCTVNFTLFKARMFVGGFEHRFSAACNWTRGERCNIEDR

DRSEQQPLLHSTTELAILPCSFTPMPALSTGLIHHHQDIVDVQYLYGVGSGMVGWALKWEFVVLIFLLLA

DARVCVALWLMLMISQAEA

>gi|410069931|gb|AFV59187.1| envelope glycoprotein E2, partial [Hepatitis C virus]

QTHVTGGSAAREARGFTSLFSLGASQKLQLVNTNGSWHINRTALNCNESINTGFIAGLFYYHKFNSTRCP

QRLSSCKPITSFEQGWGPITDANISGSSNDRPYCWHYAPRPCGVVNASSVCGPVYCFTPSPVVVGTTDAK

GVPTYTWGENETDVFLLESLRPPGGRWLGCAWMNSTGFLKTCGAPPCDIYGGSKKPDNNQALFCPTDCFR

KHPDATYSRCGAGPWITPRCMVDYPYRLWHYPCTVNFTLFKVRMFVGGFEHRFSAACNWTRGERCNIEDR

DRSEQQPLLHSTTEFAILPCSFTPMPALSTGLIHLHQDIVDVQYLYGVGSGMVGWALKWEFVVLIFLLLA

DARVCVALWLMLMISQAEA

>gi|410069927|gb|AFV59185.1| envelope glycoprotein E2, partial [Hepatitis C virus]

QTHVTGGSAAREARGFTSLFSLGASQKLQLVNTNGSWHINRTALNCNESINTGFIAGLFYYHKFNSTGCP

QRLSSCKPITSFEQGWGPITDANISGSSNDRPYCWHYAPRPCGVVNASSVCGPVYCFTPSPVVVGTTDAK

GVPTYTWGENETDVFLLESLRPPGGRWLGCAWMNSTGFLKTCGAPPCDIYGGSKKPDNNQALFCPTDCFR

KHPDATYSRCGAGPWITPRCMVDYPYRLWHYPCTVNFTLFKVRMFVGGFEHRFSAACNWTRGERCNIEDR

DRSEQQPLLHSTTEFAILPCSFTPMPALSTGLIHLHQDIVDVQYLYGVGSGMVGWALKWEFVVLIFLLLA

DARVCVALWLMLMISQAEA

>gi|410069923|gb|AFV59183.1| envelope glycoprotein E2, partial [Hepatitis C virus]

QTHVTGGSAAREARGFTSLFSLGASQKLQLVNTNGSWHINRTALNCNESINTGFIAGLFYYHKFNPTGCP

QRLSSCKPITSFEQGWGPITDANISGSSNDRPHCWHYAPRPCGVVNASSVCGPVYCFTPSPVVVGTTDAK

GVPTYTWGENETDVFLLESLRPPGGRWLGCAWMNSTGFLKTCGAPPCDIYGGSKKPDNNQALFCPTDCFR

KHPDATYSRCGAGPWITPRCMVDYPYRLWHYPCTVNFTLFKVRMFVGGFEHRFSAACNWTRGERCNIEDR

DRSEQQPLLHSTTEFAILPCSFTPMPALSTGLIHLHQDIVDVQYLYGVGSGMVGWALKWEFVVLIFLLLA

DARVCVALWPMLMISQAEA

>gi|410069919|gb|AFV59181.1| envelope glycoprotein E2, partial [Hepatitis C virus]

QTHVTGGSAAREARGFTSLFSLGASQKLQLVNTNGSWHINRTALNCNESINTGFIAGLFYYHKFNSTGCP

QRLSSCKPITSFEQGWGPITDANISGSSNDRPYCWHYAPRPCGVVNASSVCGPVYCFTPSPVVVGTTDAK

GVPTYTWGENETDVFLLESLRPPGGRWLGCAWMNSTGFLKTCGAPPCDIHGGSKKPDNNQALFCPTDCFR

KHPDATYSRCGAGPWITPRCMVDYPYRLWHYACTVNFTLFKVRMFVGGFEHRFSAACNWTRGERCNIEDR

DRSEQQPLLHSTTEFATLPCSFTPMPALSTGLIHLHQDIVDVQYLYGVGSGMVGWALKWEFVVLIFLLLA

DARVCVALWLMLMISQAEA

>gi|410069915|gb|AFV59179.1| envelope glycoprotein E2, partial [Hepatitis C virus]

QTHVTGGSAAREARGSTSLFSLGASQKLQLVNTNGSWHINRTALNCNESINTGFIAGLFYYHKFNSTGCP

QRLSSCKPITSFEQGWGPITDANISGSSNDRPYCWHYAPRPCGVVNASSVCGPVYCFTPSPVVVGTTDAK

GVPTYTWGENETDVFLLESLRPPGGRWLGCAWMNSTGFLKTCGAPPCDIYGGSKKPDNNQALFCPTDCFR

KHPDATYSRCGAGPWITPRCMVDYPYRLWHYPCTVNFTLFKARMFVGGFEHRFSAACNWTRGERCNIEDR

DRSEQQPLLHSTTELAILPCSFTPMPALSTGLIHHHQDTVDVQYLYGVGSGMVGWALKWEFVVLIFLLLA

DARVCVALWLMLMISQAEA

>gi|410069911|gb|AFV59177.1| envelope glycoprotein E2, partial [Hepatitis C virus]

QAHVTGGSAAREARGFTSLFSLGASQKLLLVNTNGSWHINRTALNCNESINTGFIAGLFYYHKFNSTGCP

QRLSSCKPITSFEQGWGPITDANISGSSNDRPYRWHYAPRPCGVVNASSVCGPVYCFTPSPVVVGTTDAK

GVPTYTWGENETDVFLLESLRPPGGRWLGCAWMNSTGFLKTCGAPPCDIYGGSKKPDNNQALFCPTDCFR

KHPDATYSRCGAGPWITPRCMVDYPYRLWHYPCTVNFTLFKVRMFVGGFEHRFSAACNWTRGERCNIEDR

DRSEQQPLLHSTTEFAILPCSFTPMPALSTGLIHLHQDIVDVQYLYGVGSGMVGWALKWEFVVLIFLLQA

DARVCVALWLMLMISQAEA

>gi|323361110|gb|ADX42056.1| polyprotein [Hepatitis C virus subtype 3a]

GTHITGDSAGSTVRSVVGLFNPGAQQNLQLVNTNGSWHINRTALNCDESINTGFLAGLFYHNKFNSSGCP

QMLSSCKPITFFKQGWGPLTDANITDTSNDRPYCWHYAPRPCKTVKALDVCGPVYCFTPSPVVVGTTDAK

GAPTYTWGENETDVFLLESLRPPSGRWFGCSWMNSTGFLKTCGAPPCNIYGAGEAPGNGSGLFCPTDCFR

KHPEATYSRCGAGPWLTPRCIVDYPYRLWHYPCTVNFTLFKVRMFVGGFEHRFDAACNWTRGERCDIEDR

DRSEQHPLLHSTTELAILPCSFTPMPALSTGLIHLHQNIVDVQYLYGVGSGMVGWALKWEFVILVFLLLA

DA

>gi|323361106|gb|ADX42054.1| polyprotein [Hepatitis C virus subtype 3a]

STYTSGGSAARDVHGLVGLFSPGAQQRLQLVNTNGSWHINRTSLNCNDSINTGFIAGLFYHHKFNSTGCP

QRLSSCKPITFFKQGWGPLTDANITGSSNDRPYCWHYPPRPCEVVRASSVCGPVYCFTPSPVVVGTTDSR

GAPTYRLGENETDVFLLTSLRPPAGRWFGCTWMNSTGFVKTCGAPPCNIYGGEEGRNPHNESDLFCPTDC

FRKHPEATYSRCGAGPWLTPRCLVDYPYRLWHYPCTVNFTLFKVRMFVAGFEHRFQAACNWTRGERCDIE

DRDRSELHPLLHSTTELAILPCSFTPMPALSTGLIHLHQNIVDVQYLYGVGAGMVGWAVKWEFVILVFLL

LADA

>gi|323361102|gb|ADX42052.1| polyprotein [Hepatitis C virus subtype 3a]

STYTSGGSAARDVHGLVGLFSPGAQRRLQLVNTNGSWHINRTSLNRNDSINTGFIAGLFYHHKFNSTGCP

QRLSSCKPITFFKQGWGPLTDANITGSSNDRPYCWHYPPRPCEVVRASSVCGPVYCFTPSPVVVGTTDSR

GAPTYRLGENETNVFLLTSLRPPAGRWFGCTWMNSTGFVKTCGAPPCNIYGGEEGRNPHNESDLFCPTDC

FRKHPEATYSRCGAGPWLTPRCLVDYPYRLWHYPCTVNFTLFKVRMFVAGFEHRFQAACNWTRGERCDIE

DRDRSELHPLLHSTTELAILPCSFTPMPALSTGLIHLHQNIVDVQCLYGVGAGMVGRAVKWEFVILVFLL

LADA

>gi|323361098|gb|ADX42050.1| polyprotein [Hepatitis C virus subtype 3a]

NTYVTGGKAASTTSGLVSLFSRGASQSLQLIETNGSWHINRTALNCNDSINTGFVAGLFYYHRFNSTGCP

GRLSSCKPLTYFKQGWGPLTDANISGSSDDRPYCWHYPPRPCGIVPASSVCGPVYCFTPSPVVVGTTDDK

GVPTYTRGENETDVFLLTSCGPPAGRWFGCTWMNSTGFTKTCGAPPCNIYGVRERGSRNETDLICPTDCF

RKHPGATYSRCGTGPWLTPRCLVDYPYRLWHYPCTVNFTLFKVRMFIGGFEHRFQAACNWTRGERCDIED

RDRSEQHPLLHSTTGLAILPCSFTPMPALSTGLIHLHQNIVDVQYLYGVGAGMVGWALKWEFVILVFLLL

ADA

>gi|323361094|gb|ADX42048.1| polyprotein [Hepatitis C virus subtype 3a]

NTYVTGGKAASTTSGLVSLFSRGASQNLQLIETNGSWHINRTALNCNDSINTGFIAGLFHYHRFNSTGCP

GRLSSCKPLTYFKQGWGPLTDANISGSSDDRPYCWHYPPRPCGIVPASSVCGPVYCFTPSPVVVGTTDDK

GVPTYTLSENETDVFLLTSCGPPAGRWFGCTWMNSTGFTKTCGAPPCNIYGVRERGSRNETDLICPTDCF

GKHPGATYSRCGAGPWLTPRCLVGYPYRLWHYPCTVNFTLFKVRMFIGGFEHRFQAACNWTRGERCDIGD

RDRSEQHPLLHSTTELAILPCSFTPMPALSTGLIHLHQNIVDVQYLYGVGAGMVGWALKWEFVILVFLLL

ADA

>gi|323361090|gb|ADX42046.1| polyprotein [Hepatitis C virus subtype 3a]

NTYVTGGKAASTTSGLVSLFSRGASQNLQLIETNGSWHINRTALNCNDSINTGFIAGLFYYHRFNSTGCP

GRLSSCKPLTYFKQGWGPLTDANISGSSDDRPYCWHYPPRPCGIVPASSVCGPVYCFTPSPVVVGTTDDK

GVPTYTWGENETDVFLLTSCGPPAGRWFGCTWMNSTGFTKTCGAPPCNIYGVRERGFRNETDLICPTDCF

RKHPGATYSRCGAGPWLTPRCLVDYPYRLWHYPCTVNFTLFKVRMFIGGFEHRFQAACNWTRGERCDIED

RDRSEQHPLLHSTTELAILPCSFTLMPALSTGLIHLHQNIVDVQYLYGVGAGMVGWALKWEFVILVFLLL

ADA

>gi|323361086|gb|ADX42044.1| polyprotein [Hepatitis C virus subtype 3a]

GTYVTGGQAASHTKGFTNLFSAGPKQNLQLVNTNGSWHINSTALNCSESINTGFLAGLFYYHRFNSTGCP

ARLSSCRPLSSFRQGWGPLTDANISNPFFDRPYCWHYPPRPCGTIPALGVCGPLYCFTSSPVVVGTTDSS

GAPTYSWGENETDVFLLESLRPPGGRWFGCVWMNSTGFLKPCGAPPCNIYGGGGELENETDLFCPTDCFR

KHPEATYSRCGAGPWLTPRCMADYPYRLWHYPCTVNFTLFKVRMFVGGFEHRFSAACNWTRGERCDIEDR

DRSEQHPLLHSTTELAILPCSFTPMPALSTGLIHLHQNIVDVQYLYGVGSGMMGWALKWEVVILVFLLLA

DA

>gi|323361082|gb|ADX42042.1| polyprotein [Hepatitis C virus subtype 3a]

GTYVTGGQAASHTKGFTNLFSAGPKQNLQLVNTNGSWHINSTALNCNESINTGFLAGLFYYHRFNSTGCP

ARLSSCRPLSSFRQGWGPLTDANISNPFFDRPYCWHYPPRPCALIPALGVCGPVYCFTPSPVVVGTTDSS

GAPTYSWGENETDVFLLESLRPPGGRWFGCVWMNSTGFLKTCGAPPCNIYGGGGSLENETDLFCPTDCFR

KHPEATYSRCGAGPWLTPRCMVDYPYRLWHYPCTVNFTLFKMRMFVGGFEHRFSAACNWTRGERCDIEDR

DRSEQHPLLHSTTELAILPCSFTPMPALSTGLIHLHQNIVDVQYLYGVGSGMVGWALKWEVVILVFLLLA

DA

>gi|323361078|gb|ADX42040.1| polyprotein [Hepatitis C virus subtype 3a]

STYTSGGSAARDVHGLVGLFSPGAQQRLQLVNTNGSWHINRTSLNCNDSINTGFIAGLFYHHKFNSTGCP

QRLSSCKPITFFKQGWGPLTDANITGPSNDRPYCWHYPPRPCEVVRASSVCGPVYCFTPSPVVVGTTDSR

GAPTYSWGENETDVFLLTSLRPPAGRWFGCTWMNSTGFVKTCGAPPCNIYGGEEARNPHNESDSFCPTDC

FRKHPEATYSRCGAGPWLTPRCLVDYPYRLWHYPCTVHFTLFKVRMFVAGFEHRFQAACNWTRGERCDIE

DRDRSELHPLLHSTTELAILPCSFTPMPALSTGLIHLHQNIVDVQYLYGVGAGMVGWAVKWEFVILVFLL

LADA

>gi|323361074|gb|ADX42038.1| polyprotein [Hepatitis C virus subtype 3a]

STYTSGGSAARDVHGLVGLSSPGAQQRLQLVNTNGSWHINRTSLNCNDSINTGFIAGLFYHHKFNSTGCP

QRLSSCKPITFFKQGWGPLTDANITGPSNDRPYCWHYPPRPCEVVRASSVCGPVYCFTPSPVVVGTTDVG

GAPTYSWGENETDVFLLTSLRPPAGRWFGCTWMNSTGFVKTCGAPPCNIYGGEEARNPHNESDSFCPTDC

FRKHPEATYSRCGAGPWLTPRCLVDYPYRLWHYPCTVNFTLFKVRMFVAGFEHRFQAACNWTRGERCDIE

DRDRSELHPLLHSTTELAILPCSFTPMPALSTGLIHLHQNIVDVQYLYGVGASVVGWAVKWEFVILVFLL

LADA

>gi|323361070|gb|ADX42036.1| polyprotein [Hepatitis C virus subtype 3a]

STYTSGGSAARDIHGLVGLFSPGAQQRLQLVNTNGSWHINRTSLNCNDSINTGFIAGLFYHHKFNSTGCP

QRLSSCKPITFFKQGWGPLTDANITGYSNDRPYCWHYPPRPCEVVRASSVCGPAYCFTPSPVVVGTTDVG

GAPTYSWGENETDVFLLTSLRPPAGRWFGCTWMNSTGFVKTCGAPPCNIYGGEEARNPHNESDSFCPTDC

FRKHPEATYSRCGAGPWLTPRCLVDYPYRLWHYPCTVNFTLFKVRMFVAGFEHRFQAACNWTRGERCDIE

DRDRSELHPLLHSTTELAILPCSFTPMPALSTGLIHLHQNIVDVQYLYGVGAGMVGWAVKWEFVILVFLL

LADA

>gi|323361108|gb|ADX42055.1| polyprotein [Hepatitis C virus subtype 3a]

ETHITGDSAAGTVSSLVGLFNPGAQQNLQLVNTNGSWHINRTALNCDESINTGFLAGLFYHNKFNSSGCP

QMLSNCKPITFFKQGWGPLTDANITDTSNDRPYCWHYAPRPCKTVKALDVCGPVYCFTPSPVVVGTTDAK

GAPTYTWGENETDVFLLESLRPPSGRWFGCPWMNSTGFLKTCGAPPCNIYGAGEAPGNGSGLFCPTDCFR

KHPEATYSRCGAGPWLTPRCMVDYPYRLWHYPCTVNFTLFKVRMFVGGFEHRFDAACNWTRGERCDIEDR

DRSEQHPLLHSTTELAILPCSFTPMPALSTGLIHLHQNIVDVQYLYGVGAGVVGWALKWEFVILVFLLLA

DA

>gi|323361104|gb|ADX42053.1| polyprotein [Hepatitis C virus subtype 3a]

STYTSGGSAARDVHGLVGLFSPGAQRRLQLVNTNGSWHINRTSLNRNDSINTGFIAGLFYHHKFNSTGCP

QRLSSCKPITFFKQGWGPLTDANITGSSNDRPYCWHYPPRPCEVVRASSVCGPVYCFTPSPVVVGTTDSR

GVPTYRLGENETDVFLLTSLRPPAGRWFGCTWMNSTGFVKTCGAPPCNIYGGEEGRNPHNESDLFCPTDC

FRKHPEATYSRCGAGPWLTPRCLVDYPYRLWHYPCTVNFTLFKVRMFVAGFEHRFQAACNWTRGERCDIE

DRDRSELHPLLHSTTELAILPCSFTPMPALSTGLIHLHQNIVDVQCLYGVGAGMVGRAVKWEFVILVFLL

LADA

>gi|323361100|gb|ADX42051.1| polyprotein [Hepatitis C virus subtype 3a]

STYTSGGSAARDVHGLVGLFSPGAQQRLQLVNTNGSWHINRTSLNCNDSINTGFIAGLFYHHKFNSTGCP

QRPSSCKPITFFKQGWGPLTDANITGSSNDRPYCWHYPPRPCEVVRASSVCSPVYCFTPSPVVVGTTDSR

GVPTYTLGENETDVFLLTSLRPPCGRWFGCTWMNSTGFVKTCGAPPCNIYGGEEGRNPHNESDLFCPTDC

FRKHPEATYSRCGAGPWLTPRCLVDYPYRLWHYPCTVNFTLFKVRMFVAGFEHRFQAACNWTRGERCDIE

DRDRSELHPLLHSTTELAILPCSFTPMPALSTGLIHLHQNIVDVQYLYGVGAGMVGWAVKWEFVILVFLL

LADA

>gi|323361096|gb|ADX42049.1| polyprotein [Hepatitis C virus subtype 3a]

NTYVTGGKAASTTSGLVSLFSRGASQNLQLIETNGSWHINRTALNCNDSINTGFIAGLFYYHRFNSTGCP

GRLSSCKPLTYFKQGWGPLTDANISGSSDDRPYCWHYPPRPCGIVPASSVCGPVYCFTPSPVVVGTTNDK

GVPTYTWGENETDVFLLTSCGPPAGRWFGCTWMNSTGFTKTCGAPPCNIYGVRERGSRNETDLICPTDCF

RKHPGATYSRCGAGPWLTPRCLVDYPYRLWHYPCTVNFTLFKVRMFIGGFEHRFQAACNWTRGERCDIED

RDRSEQHPLLHSTAELAILPCSFTPMPALSTGLIHLHQNIVDVQYLYGVGAGMVGWALKWEFVILVFLLL

ADA

>gi|323361092|gb|ADX42047.1| polyprotein [Hepatitis C virus subtype 3a]

NTYVTGGKAASTTSGLVSLFSRGASQNLQLIETNGSWHINRTALNCNDSINTGFIAGLFYYHRFNSTGCP

GRLSSCKPLTYFKQGWGPLTDANISGSSDDRPYCWHYPPRPWGIVPASSVCGPVYCFTPSPVVVGTTDDK

GVPTYTRGENETDVFLLTSCGPPAGRWFGCTWMNSTGFTKTCGAPPCNIYGVRERGSRNETDLICPTDCF

RKHPGATYSRCGAGPWLTPRCLVDYPYRLWHYPCTVNFTLFKVRMFIGGFEHRFQAACNWTRGERCDIED

RDRSEQHPLLRSTTELAILPCSFTPMPALSTGLIHLHQNIVDVQYLYGVGAGMVGWALKWEFVILVFLLL

ADA

>gi|323361088|gb|ADX42045.1| polyprotein [Hepatitis C virus subtype 3a]

GTYVTGGQAASHTKGFTNLLSAGPKQNLQLVNTNGSWHINSTALNCNESINTGFLAGLFYYHRFNSTGCP

ARLSSCRPLSSFRQGWGPLTDANISNPFFDRPYCWHYPPRPCGTIPALGVCGPVYCFTPSPVVVGTTDSS

GAPTYSWSENETDVFLLESLRPPRGRWFGCVWMNSTGFLKTCGAPPCNIYGGGGKLENETDLFCPTDCFR

KHPEATYSRCGAGPWLTPRCMVDYPYRLWHYPCTVNFTLFKVRMFVGGFEHRFSAACNWTRGERCDIEDR

DRSEQHPLLHSTTELAILPCPFTPMPALSTGLIHLHQNIVDVQYLYGVGSGMVGWALKWEVVILVFLLLA

DA

>gi|323361084|gb|ADX42043.1| polyprotein [Hepatitis C virus subtype 3a]

GTYVTGGQAASHTKGFTNLFSAGPKQNLQLVNTNGSWHINSTALNCNESINTGFLAGLFYYHRFNSTGCP

ARLSSCRPLSSFRQGWGPLTDANISNPFFDRPYCWHYPPRPCGTIPALGVCGPVYCFTPSPVVVGTTDSS

GAPTYSWGENETDVFLLESLRPPGGRWLGACGMNSTGFLKTCEAPPCNIYGGGGSLENETDLFCPTDCFR

KHPEATYSRCGAGPWLTPRCMVDYPYRLWHYPCTVNFTLFKVRMFVGGFEHRFSAACNWTRGERCDIEDR

DRSEQHPLLHSTTELAILPCSFTPMPALSTGLIHLHQDIVDVQYLYGVGSGMVGWALKWEVVILVFLLLA

DA

>gi|323361080|gb|ADX42041.1| polyprotein [Hepatitis C virus subtype 3a]

GTYVTGGQAASHTKGFTNLFSAGPKQNLQLVNTNGSWHINSTALNCNESINTGFLAGLFYYHRFNSTGCP

ARLSSCRPLSFFRQGWGPLTDANISNPFFDRPYCWHYPPRPCALIPALGVCRPLYCFTPSPVVVGTTDSR

GAPTYTWSENETDVFLLESLRPPRGRWFGCVWMNSTGFLKPCGAPPCSIYGGGGKLENETDLFCPTDCFR

KHPEATYSRCGAGPWLTPRCMVDYPYRLWHYPCTVNFTLFKVRMFVGGFEHRFSAACNWTRGERCGIEDR

DRSEQHPLLHSTTELAILPCSFTPMPALSTGLIHLHQNIVDVQYLYGVGSGMVGWALKWEVVILVFLLLA

DA

>gi|323361076|gb|ADX42039.1| polyprotein [Hepatitis C virus subtype 3a]

STYTSGGSAARDVHGLVGLFSPGAQQRLQLVNTNGSWHINRTSLNCNDSINTGFIAGLFYHHKFNSTGCP

QRLSSCKPITFFKQGWGPLTDANITGSSNDRPYCWHYPPRPCEVVRASSVCGPVYCFTPPPVVVGTTDSR

GAPTYSWGENETDVFLLTSLRPPAGRWFGCTWMNSTGFVKTCGAPPCNIYGGEEAGNPHNESDSFCPTDC

FRKHPEATYSRCGAGPWLTPRCLVDYPYRLWHYPCTVNFTLFKVRMFVAGFEHRFQAACNWTRGERCDIE

DRDRSELHPLLHSTTELAILPCSFTPMPALPTGLIHLHQNIVDVQYLYGVGAGMVGWAVKWEFVILVFLL

LADA

>gi|323361072|gb|ADX42037.1| polyprotein [Hepatitis C virus subtype 3a]

STYTSGGSAARDVHGLVGLFSPGAQQRLQLVNTNGSWHINRTSLNCNDSINTGFTAGLFYYHKFNSIGCP

QRLSSCKPITFFKQGWGPLTDANITGSSNDRPYCWHYPPRPCEVVRASSVCGPVYCFTPSPVVVGTTDVV

GAPTYSWGENETDVFLLTSLRPPAGRWFGCMWMNSTGFVKTCGAPPCNIYGGEEARNPHNESDSFCPTDC

FRKHPEATYSRCGAGPWLTPRCLVDYPYRLWHYPCTVNFTLFKVRMFVAGFEHRFQAACNWTRGERCDIE

DRDRSELHPLLHSTTELAILPCSFTPMPALSTGLIHLHQNIVDVQYLYGVGAGMVGWAVKWEFVILVFLL

LADA

>gi|339188997|gb|AEJ35163.1| envelope protein E2 [Hepatitis C virus]

STYTSGGSAARDIHGLVGLFSPGAQQRLQLVNTNGSWHINRTSLNCNDSINTGFIAGLFYHHKFNSTGCP

QRLSSCKPITFFKQGWGPLTDANISGSSDDKPYCWHYAPRSCNTVPASSVCGPVYCFTPSPVVVGTTDAK

GVPTYNWGENETDVFLLESCGPPSGRWFGCVWMNSTGFLKTCGAPSCDIYGGNGRRGNDTDLFCPTDCFR

KHPEATYSRCGAGHWLTPRCMVDYPYRLWHYPCTVNFTLFKVRMFVGGFEHRLSAACNWTRGERCDIEDR

DRSEQHPLLHSTTELAILPCSFTPMPALSTGLIHLHQNIVDVQYLYGVGSGVVGWAVKWEFVILVFLLLA

DA

>gi|310975705|gb|ADP55198.1| envelope protein E2 [Hepatitis C virus]

VTHTVGGSVARSTSSLTSLFSPGASQNLQLINTNGSWHINRTALNCNDSINTGFIAGLIYRYRFNSTGCP

SMLSSCKPVTFFNQGWGPLTDGNISGPSDDKPYCWHYAPRSCSTVPASSVCGPVYCFTPSPVVVGTTDAK

GVPTYNWGENETDVFLLESCGPPSGRWLGCVWMNSTGFLKTCGAPSCDIYGGNGRRGNDTDLFCPTDCFR

KHPEATYSRCGAGPWLTPRCMVDYPYRLWHYPCTVSFTLFKVRMFVGGFEHRLSAACNWTRGERCDIEDR

DRSEQHPLLHSTTELAILPCSFTPMPALSTGLIHLHQNIVDVQYLYGVGSGVVGWAVKWEFVILVFLLLA

DA

>gi|310975701|gb|ADP55196.1| envelope protein E2 [Hepatitis C virus]

VTHTAGGSVARSTSSLTSLFSPGASQNLQLINTNGSWHINRTALNCNDSINTGFIAGLIYRYRFNSTGCP

SMLSSCKPITFFNQGWGPLTDGNISGPSDDKPYCWHYAPRSCSTAPASKVCGPVYCFTPSPVVVGTTDAK

GVPTYTWGENETDGFLLESCGPPSGRWFGCVWMNSTGFLKTCGAPSCDIYGGNGRRGNDTDLFCPTDCFR

KHPEATYSRCGAGPWLAPRCMVDYPYRLWHYPCTVNFTLFKVRMFVGGFEHRLSAACNWTRGERCDIEDR

DRSEQHPLLHSTTELAILPCSFTPMPALSTGLIHLHQNIVDVQYLYGVGSGVVGWAVKWEFVILVFLLLA

DA

>gi|310975697|gb|ADP55194.1| envelope protein E2 [Hepatitis C virus]

KTYITGSNIAHNTRGLASLFTSGAKQKLQLVNTNGSWHINSTALNCNDSLNTGFLAGLLYHHKFNSTGCP

QRLSSCKPLAFFRQGWGSLTDANITGPSNDRPYCWHYAPRPCGIVPALNVCGPVYCFTPSPVVVGTTDAR

GAPTYTWGENETDVFLLKSCRPPRGRWFGCVWMNSTGFLKTCGAPPCNIYEVGGIPNNESDLFCPTDCYR

KHPEATYSRCGAGPWLTPRCMVDYPYRLWHFPCTVNFTLFKVRMFVGGFEHRFSAACNWTRGERCDIEDR

DRSEQQPLLHSTTELAILPCSFTPMPALSTGLIHLHQNIVDVQYLYGVGSGMVGWALKWEFVILVFLLLA

DA

>gi|310975707|gb|ADP55199.1| envelope protein E2 [Hepatitis C virus]

VTHTVGGSVARSTSSLTSLLSPGASQNLQLINTNGSWHINRTALNCNDSINTGFIAGLIYRYRFNSTGCP

SMLSSCKPITFFNQGWGPLTDGNISGPSDDKPYCWHYAPRSCSTVPASSVCGPVYCFTPSPVVVGTTDAK

GVPTYNWGENETDVFLLESCGPPSGRWFGCVWMNSTGFLKTCGAPSCDIYGGNGRRGNDTDLFCPTDCFR

KHPEATYSRCGAGPWLTPRCMVDYPYRLWHYPCTVNFTLFKVRMFVGGFEHRLSAACNWTRGERCDIEDR

DRSEQHPLLHSTTELAILPCSFTPMPALSTGLIHLHQNIVDVQYHYGVGSGVVGWAVKWEFVILVFLLLA

DA

>gi|310975703|gb|ADP55197.1| envelope protein E2 [Hepatitis C virus]

VTHTVGGSVARSTSSLTSLFSPGASQNLQLINTNGSWHINRTALNCNDSINTGFIAGLIYRYRFNSTGCP

SMLSSCKPITFFDQGWGPLTDGNISGPSDDKPYCWHYAPRSCSTVPASRVCGPVYCFTPSPVVVGTTEAK

GVPTYNWGENETDVFLLESCGPPSGRWFGCVWMNSTGFLKTCGAPSCDIYGGNGRRGNDTDLFCPTDCFR

KHPEATYSRCGAGPWLTPRCMVDYPYRLWHYPCTVNFTLFKVRMFVGGFEHRLSAACNWTRGERCDIEDR

DRSEQHPLLHSTTELAILPCSFTPMPALSTGLIHLHQNIVDVQYLYGVGPGVVGWAVKWEFVILVFLLLA

DA
